# Prevalence, species identification, and antibiotic resistance of Staphylococci in dogs visiting veterinary clinics in Vietnam

**DOI:** 10.1371/journal.pone.0328472

**Published:** 2025-07-24

**Authors:** Nguyen Thi Lan Anh, Nguyen Vu Thuy Hong Loan, Nguyen Thuy Y Vi, Dao Huyen Tran, Luu Thi Thanh Hang, Sandra Steele, Lam Thanh Nguyen

**Affiliations:** 1 Faculty of Veterinary Medicine and Animal Husbandry, HUTECH University, Thanh My Tay ward, Ho Chi Minh city, Vietnam; 2 Faculty of Veterinary Medicine, College of Agriculture, Can Tho University, Ninh Kieu ward, Can Tho city, Vietnam; 3 Melbourne Veterinary School, The University of Melbourne, Parkville, Victoria, Australia; Suez Canal University, EGYPT

## Abstract

Staphylococci are important commensal and opportunistic bacteria found in various animals, including dogs and humans. The emergence of antibiotic-resistant Staphylococci is a growing global concern, including in Vietnam. This study aimed to investigate the prevalence, species distribution, and antibiotic-resistance profiles of Staphylococci isolated from dogs visiting veterinary clinics in Vietnam. A total of 309 *Staphylococcus* strains were isolated from 410 nasal and skin samples collected from both healthy and diseased dogs between December 2021 and December 2023 in Ho Chi Minh city. The isolation rate of *Staphylococcus* spp. was 71.2% (95% confidence interval [CI]: 66.6%–75.6%), with 78.9% (95% CI: 73.6%–83.7%) in diseased dogs, 56.9% (95% CI: 48.4%–65.2%) in healthy dogs, 80.1% (95% CI: 74.3%–85.1%) in skin samples, and 60.3% (95% CI: 52.9%–67.5%) in nasal samples. Species identification indicated that *S. pseudintermedius* was dominant, followed by *S. aureus*. Other species identified included *S. epidermidis* and *S. schleiferi.* Antibiotic susceptibility testing showed complex resistance patterns. Approximately 91.3% of isolates were resistant to at least one antibiotic, and 60.5% were multidrug-resistant (resistant to three or more antibiotics). A total of 215 antibiotic-resistance phenotypes were observed, with 85 phenotypes showing resistance to more than ten different antibiotics. Isolates from diseased dogs exhibited higher antibiotic-resistance rates than those from healthy dogs. Several antibiotic-resistance genes were identified, with *aacA-aphD* being the most prevalent, followed by *tetK*, *gyrA*, *mecA*, *msrA*, *dfrA*, and *ermA*. These findings highlight the widespread presence of antibiotic-resistant Staphylococci in dogs and emphasize the necessity for ongoing surveillance of antibiotic-resistance evolution in animals and its implications for human health.

## 1. Introduction

*Staphylococcus* is a Gram-positive aerobic genus with more than 90 species and 30 subspecies [1]. These organisms are common commensals and opportunistic pathogens of the skin and mucosal membranes, temporarily colonizing the intestinal tract in humans and other animals [2]. Based on their ability to coagulate rabbit plasma, Staphylococci are categorized into two groups, coagulase-positive (CoPS) and coagulase-negative (CoNS) [1]. CoPS are well recognized as important pathogens; *Staphylococcus aureus* (*S. aureus*) is a primary or opportunistic pathogen in humans, and *Staphylococcus pseudintermedius* (*S. pseudintermedius*) is commonly found in canine microbiota and infections [3–5]. CoNS are part of the normal microbiota of humans and animals; however, they have been implicated in certain serious infections [6].

Globally, the impacts of antimicrobial resistance on human and animal health are increasing [7–9]. *Staphylococcus* spp. are capable of developing antibiotic resistance, including multidrug resistance (MDR), and can serve as reservoirs of drug resistance genes [10]. In particular, the emergence and spread of methicillin-resistant *S. aureus* (MRSA) has important ramifications on public health [11]. Additionally, several other *Staphylococcus* spp. have also developed methicillin resistance [12]. Resistance to linezolid, an antimicrobial effective against MDR Gram-positive pathogens, has been discovered in *Staphylococcus spp.* in dogs. Several studies in dogs revealed that *Staphylococcus* isolates often exhibit MDR [8,13], including resistance to beta-lactams, clindamycin, tetracycline, fluoroquinolones, and trimethoprim-sulfamethoxazole [14–16].

Transmission of *S. aureus* between humans and dogs has been reported in several countries [17,18], with dogs acting as reservoirs for some cases. Dogs are natural carriers of *S. pseudintermedius,* with the bacterium being frequently isolated from the nostrils, oropharynx, and perianal region. Staphylococci have progressively acquired MDR during adaptation and have been identified as zoonotic pathogens, particularly posing risks to immunocompromised individuals [19–21]. Therefore, monitoring Staphylococci and antimicrobial resistance is essential for epidemiological studies, enabling the tracking of species distribution and the evolution of antibiotic resistance across spatial and temporal contexts. This knowledge supports formulating infection control strategies, informs antibiotic stewardship efforts, and strengthens coordinated interventions in veterinary and human healthcare sectors. Ultimately, such measures are crucial in mitigating the broader impact of antimicrobial resistance [22].

In Vietnam, concerns regarding MDR *Staphylococcus* spp. have gained prominence. MDR *S*. *aureus* showed a prevalence rate of 51.8% in children [23], with 13.39% of those with pneumonia being infected with MRSA [24]. In adults, the prevalence was 76.5% [25], with more than 63 MRSA isolates and seven methicillin-resistant coagulase-negative S*taphylococcus* (MRCoNS) strains detected in Ho Chi Minh city [26]. The MRCoNS strains (*S. haemolyticus* and *S. cohnii*) displayed intermediate resistance to linezolid and carried the *cfr* gene. *S. aureus* was recently recognized to be responsible for 15.8% of the bloodstream infections detected in a teaching hospital, with MDR being identified in 57.0% of the cases [9].

The current understanding of the distribution and antimicrobial resistance patterns of *Staphylococcus* spp. in domestic animals in Vietnam is limited. Existing studies on MRSA in production animals have shown a 59.6% (59/99) prevalence in healthy and diseased pigs [27] and 1.5% (6/400) in raw milk of cows with subclinical mastitis [28]. However, studies in pet animals, such as dogs, which are in close contact with humans, are lacking. This study aimed to address these critical research gaps by (*i*) investigating the prevalence and species distribution of *Staphylococci* and (*ii*) determining the antibiotic susceptibility of the bacteria isolated from healthy and diseased dogs visiting veterinary clinics in Vietnam.

## 2. Materials and methods

### 2.1. Ethics approval

All experimental protocols were approved by the Institutional Animal Care and Use Committee of Can Tho University (ethics approval No. DT2021-04/KNN). Swab sampling was performed according to the guidelines in the Regulation on Animal Experimentation of Can Tho University, Vietnam. Informed consent was obtained from all dog owners through verbal explanation of the purpose and requirements of the study.

### 2.2. Study areas, study period, and sample collection

The cross-sectional study was conducted from December 2021 to December 2023 on dogs visiting ten veterinary clinics in Ho Chi Minh city, Vietnam. The veterinary clinics are located in the central area of Ho Chi Minh city and its neighbouring districts, including Animal Health Laboratory and Treatment Division of Ho Chi Minh City. Animals that met the sampling criteria and had the owner’s consent were randomly sampled until the sample size was reached. A minimum sample size of 138 was calculated using Cochran’s formula [29]:


$$n=z^{2}x\;\frac{p(1-p)}{d^{2}}$$

where:

*n*: Sample size.

*z*: The standard normal value for the desired confidence level (z = 1.96 in this study).

*d*: The maximum difference between observed and actual infection rates (no more than 5%).

*p*: Estimated infection rate (10%).

Dogs included in this study were divided into two groups: (a) healthy dogs visiting for vaccination or routine health check-ups with no history of illness and normal clinical examination; and (b) dogs presenting to the clinics with clinical signs of respiratory disease (nasal discharge with cloudy or purulent mucus, dry or wet cough, or dyspnoea) or skin disease (itching, increased skin pigmentation or redness, foul odor, increased mucus secretion with or without pus at the inflammation site, hair loss, small pimples on the skin and scaly or keratinized skin).

Samples were taken from the two study groups, with one swab collected from either the nares or skin of each dog. In healthy dogs, an equal number of samples were collected from each of the clinics, with the sample site determined using a simple random sampling method, while in diseased dogs, samples were obtained from the site of infection. The sampling procedure of dogs with diseased skin followed the guidelines by Hillier et al (2014) [30] and was performed by veterinarians. Each sample was placed in a labelled tube containing sterile Stuart Amies solution and stored in containers at 2–8°C. The samples were immediately transported to the Laboratory of Microbiology, Faculty of Veterinary Medicine and Animal Husbandry, HUTECH University, for bacterial isolation.

#### 2.3. Identification of *Staphylococcus* species using bacterial isolation and PCR.

Each swab was streaked onto blood agar base, Baird–Parker agar (BPA) supplemented with egg yolk tellurite, and mannitol salt agar (MSA) plates (Oxoid, UK). The isolation procedure was performed following methods described in previous studies [4]. Plates were incubated aerobically at 37°C for 24 hours, and the colonies with typical morphology were chosen for further investigations. Typical morphology of colonies was determined as follows: on MSA, colonies were round, convex, yellow or white, with the surrounding area turning yellow or displaying no color shift. On BPA agar, colonies were round, convex, black, with or without a halo around them. On blood agar, colonies were round, large, milky white, with or without hemolysis. One to two colonies with different morphology were selected from each sample. These colonies were purified on nutrient agar and underwent Gram staining. Oxidase and catalase tests were then performed on these colonies. The procedures of Gram staining, oxidase and catalase tests were performed as described in Markey et al. (2013) [2]. In the case of negative oxidase and positive catalase tests, the colonies were transferred into Brain Heart Infusion broth (HiMedia Laboratories, India) supplemented with 30% glycerol and stored at −20°C for further investigations.

Staphylococci were confirmed and identified at the species level by conventional and multiplex PCR (S1 Fig). For DNA extraction, a 500-μL aliquot of each culture suspension was utilized, following the Tris-EDTA-NaCl-Triton X100 method [31]. Seven primer pairs were used in the multiplex PCR to determine *Staphylococcus* species. Multiplex PCR was carried out in a total volume of 20-μL reaction mixture, which consisted of 3 μL of genomic DNA template, 13 μL of the GoTaq^®^ Colorless Master Mix (Promega, USA), 2 μL of DEPC-treated water and 1 μL each of 10 μM forward and reverse primers as indicated in S1 Table. PCR cycling conditions were carried out as previously described [32–36] with 94°C for 4 min, followed by 35 cycles of 94°C for 60 s, and annealing temperature as described in S1 Table. Resulting PCR amplicons were electrophoresed on 1.3% tris-acetate-EDTA agarose gels stained with ethidium bromide and visualized under UV transillumination. If the PCR result was positive, a selected colony was used for antibiotic susceptibility and coagulase testing. The coagulase test followed protocols as previously described [2].

### 2.4. Antibiotic susceptibility testing

The antibiotic susceptibility of the *Staphylococcus* isolates was tested using the disk diffusion method according to the antibiotic susceptibility testing standards of the Clinical and Laboratory Standards Institute [37]. Nineteen different antibiotic agents that are widely used for treatment in humans and animals in Vietnam were tested: amoxicillin (Ax, 10 µg), penicillin (Pn, 10 IU), ampicillin (Am; 10 µg), oxacillin (Ox, 10 µg), cefoxitin (Cn, 30 µg), amoxicillin/clavulanic acid (Ac, 20/10 µg), cephalexin (Cp, 30 µg), chloramphenicol (Cl, 30 µg), ciprofloxacin (Ci, 5 µg), levofloxacin (Lv, 5 µg), clindamycin (cL, 2 µg), tetracycline (Te, 30 µg), doxycycline (Dx, 30 µg), erythromycin (Er, 15 µg), azithromycin (Az, 15 µg), gentamicin (Ge, 10 µg), amikacin (Ak, 30 µg), sulfamethoxazole/trimethoprim (Bt, 23.75 µg:1.25 µg), and linezolid (Li, 30 µg). These antibiotic agents were purchased from Nam Khoa Biotek Co. Ltd. (HCM, Vietnam). *S. aureus* subsp. *aureus* ATCC 25923 was used as a control strain for susceptibility testing to antibiotics and quality control for commercial products used in this study. Isolates were classified as MDR when they demonstrated resistance to at least three distinct antibiotic categories [38].

Minimum inhibitory concentration (MIC) of vancomycin was determined using methods described in previous studies and CLSI standards [37,39]. *Staphylococcus* spp. were cultured for 24 hours and then suspended in sterile saline with the McFarland turbidity standard 0.5. A sterile cotton swab was used to spread the suspension evenly on Mueller-Hinton agar medium. ^NK^MIC Diffusion Strips (^NK^MIC.DS) of vancomycin at concentrations ranging from 0.01 to 128 µg (Nam Khoa Biotek Co. Ltd., HCM, Vietnam) were placed on the agar surface. The MIC results were determined by the values at the intersection points between the ^NK^MIC.DS vancomycin and zone of inhibition. The susceptibility of the bacteria was read based on the MIC values according to CLSI guidelines [37].

### 2.5. Determination of antibiotic resistance genes using PCR

Conventional PCR tests were performed to detect resistance genes of the representative *Staphylococcus* isolates (S2 Fig). Seven primer pairs were used to identify antibiotic resistance genes of *Staphylococcus*, including *mecA*, *aacA*-*aphD*, *ermA*, *tetK*, *msrA*, *dfrA*, and *gyrA*, which encode resistance to beta-lactams, aminoglycosides, erythromycin, tetracycline, macrolides, trimethoprim, and fluoroquinolones, respectively. Primers and PCR conditions used to detect these genes [40–45] are described in S2 Table.

### 2.6. Statistical analysis

Data collation, processing, and initial manipulation were performed using Microsoft Excel. Aggregate data were analyzed using SPSS 22.0 statistical software. The Chi-square test (χ2) and Fisher’s exact test (when the expected frequency was less than 5) were used to compare proportions, and a *p-value* of less than 0.05 was considered statistically significant. The prevalence of *Staphylococcus* spp. isolation and the corresponding 95% confidence interval (95% CI) were calculated. Odds ratios and their 95% CI were also calculated to assess the association between the prevalence of *Staphylococcus* spp., multidrug-resistant isolates, and risk factors. Graphs and visualizations were generated using R software (version 4.3.2) with the ggplot2 package.

## 3. Results

### 3.1. *Staphylococcus* spp. distribution

**Fig 1:**
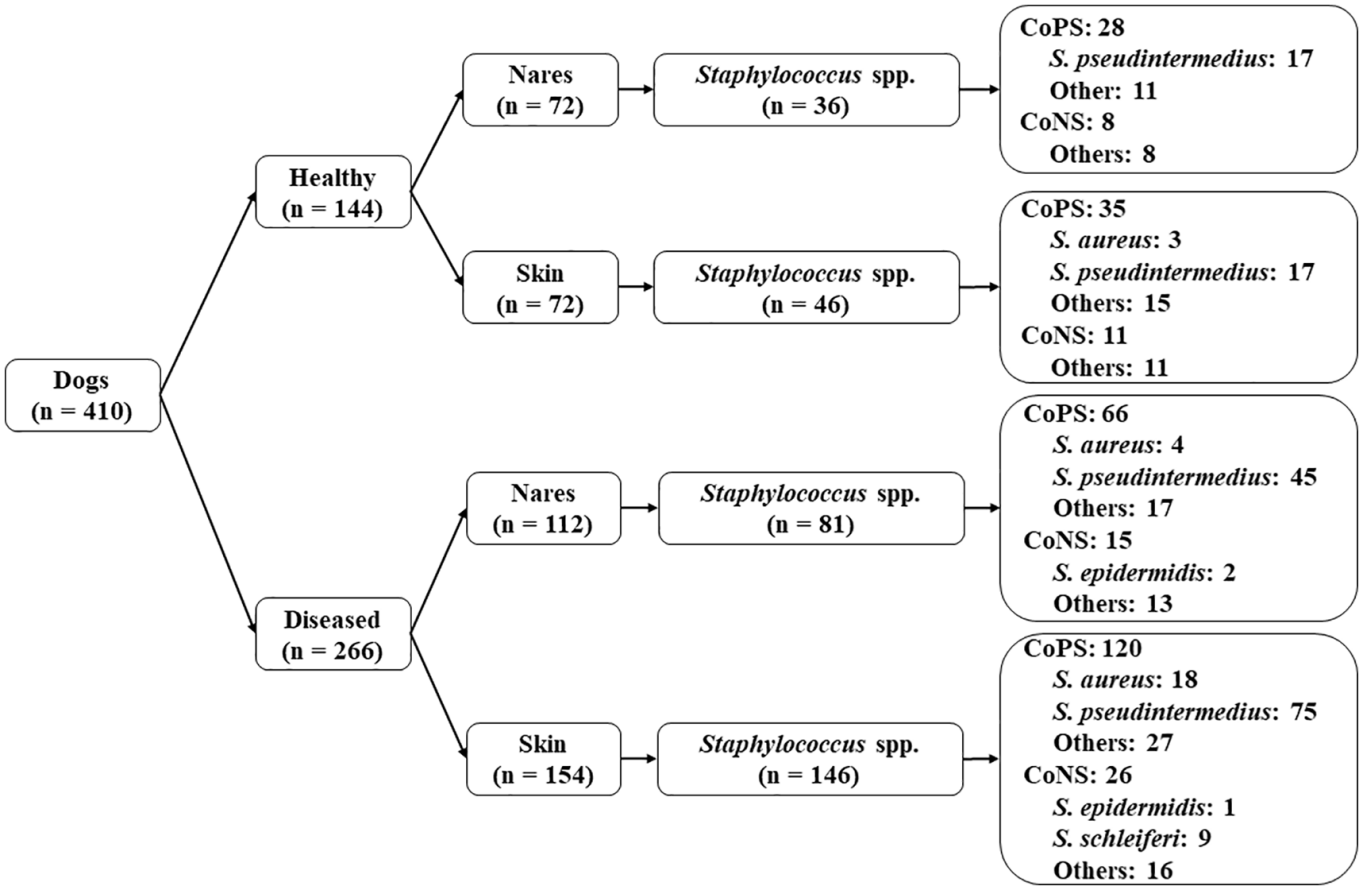
Summary of sample distribution and isolation of *Staphylococcus* spp. from 410 healthy and diseased dogs visiting the veterinary clinics in Ho Chi Minh city.

The distribution of *Staphylococcus* spp. isolated from healthy and diseased dogs in Ho Chi Minh city, Vietnam, is shown in Fig 1. The prevalence and species distribution are presented in Table 1 and S3 Table. A total of 309 *Staphylococcus* spp. were isolated from 410 dogs visiting veterinary clinics.

**Table 1:** The prevalence and species distribution of *Staphylococcus* spp. isolated from 410 healthy and diseased dogs visiting veterinary clinics in Ho Chi Minh city, Vietnam.

Sample type	No. of samples	No. of positive samples	% (95% CI)	OR (95% CI)	No. isolates	No. (%) of CoPS			No. (%) of CoNS		
						*S.* *aureus*	*S.* *pseudintermedius*	Others	*S. epidermidis*	*S.* *schleiferi*	Others
**Healthy dogs**											
Nares	72	36	50.0 (38.0–62.0)	Reference 1.77 (0.91–3.45)	36	0	17 (47.2)	11 (30.6)	0	0	8 (22.2)
Skin	72	46	63.9 (51.7–74.9)		46	3 (6.5)	17 (36.9)	15 (32.6)	0	0	11 (23.9)
Total	144	82	56.9 (48.4–65.2)		82	3 (3.7)	34 (41.5)	26 (31.7)	0	0	19 (23.2)
**Diseased dogs**											
Nares	112	75	66.9 (57.4–75.6)	Reference 3.51 (1.88–6.52)	81	4 (4.9)	45 (55.6)	17 (20.9)	2 (2.5)	0	13 (16.1)
Skin	154	135	87.7 (81.4–92.4)		146	18 (12.3)	75 (51.4)	27 (18.5)	1 (0.7)	9 (6.2)	16 (10.9)
Total	266	210	78.9 (73.6–83.7)		227	22 (9.7)	120 (52.9)	44 (19.4)	3 (1.3)	9 (3.9)	29 (12.8)
**Total**	**410**	**292**	**71.2** **(66.6–75.6)**	2.84* **(1.82–4.41)**	**309**	**25** **(8.1)**	**154** **(49.8)**	**70** **(22.7)**	**3** **(0.9)**	**9** **(2.9)**	**48** **(15.5)**

*Odds ratios and their 95% confidence intervals (CI) comparing healthy dogs (reference) to diseased dogs.

The isolation prevalence in diseased dogs was 78.9% (210 isolates/266 samples; 95% CI: 73.6%–83.7%), compared with 56.9% in healthy dogs (82 isolates/144 samples; 95% CI: 48.4%–65.2%), with OR = 2.84 (95% CI: 1.8–4.4). Among all the *Staphylococcus* isolates, 80.6% (249/309) belonged to CoPS and 19.4% (60/309) to CoNS. Additionally, four distinct *Staphylococcus* spp. were identified: *S. pseudintermedius* (154 isolates), *S. aureus* (25 isolates), *S. schleiferi* (9 isolates), and *S. epidermidis* (3 isolates). The remaining 118 isolates were of unknown species.

The distribution of *Staphylococcus* spp. across anatomical locations is summarized in Table 1 and S3 Table. A higher prevalence was observed in skin samples (181 isolates/226 samples; 80.1%; 95% CI: 74.3%–85.1%) compared with the nasal samples (111 isolates/184 samples; 60.3%; 95% CI: 52.9%–67.5%) in both healthy and diseased dogs (S3 Table). In diseased dogs, prevalence was significantly greater in skin samples (135/154; 87.7%; 95% CI: 81.4%–92.4%) than in the nasal samples (75/112 isolates; 66.9%; 95% CI: 57.4%–75.6%), with OR = 3.51 (95% CI: 1.88–6.52). Factors shown to be associated with the increased prevalence of *Staphylococcus* spp. include health status, age and management practices (S4 Table).

### 3.2. Antibiotic susceptibility

**Fig 2:**
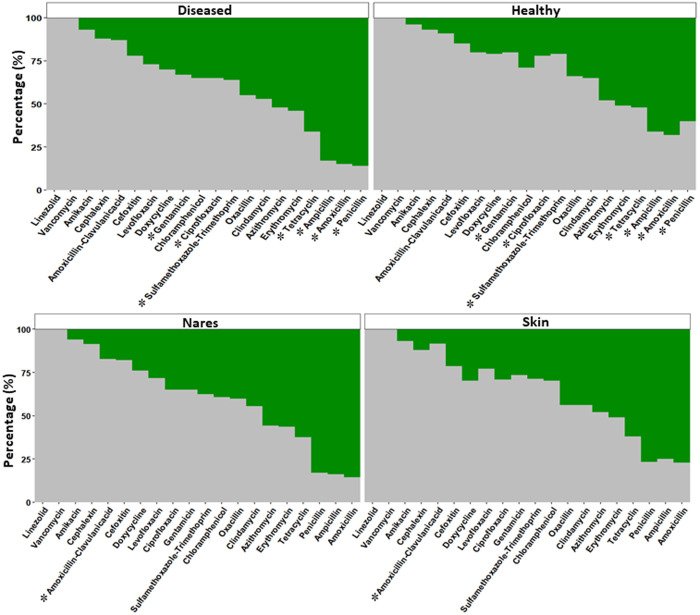
Antimicrobial susceptibility profile of 309 *Staphylococcus* spp. isolates from healthy and diseased dogs. The percentage of resistant (green) and sensitive (gray) isolates to 20 antibiotics, stratified by source: diseased and healthy dogs (upper panel) and nasal and skin samples (lower panel). (*) indicates statistically significant differences (*P* < 0.05) between groups (diseased and healthy samples, nasal and skin samples).

In total, 309 *Staphylococcus* isolates were selected for testing antibiotic susceptibility (Fig 2 and S5 Table). Approximately 91.3% (282/309 isolates) exhibited resistance to at least one antibiotic. More than 50% were resistant to amoxicillin, penicillin, ampicillin, tetracycline, erythromycin, and azithromycin (Fig 2). *Staphylococcus* spp. isolated from diseased dogs displayed a higher proportion of resistance to amoxicillin, penicillin, ampicillin, tetracycline, gentamicin, ciprofloxacin, and sulfamethoxazole-trimethoprim than those from healthy dogs. The proportion of antibiotic resistance to amoxicillin/clavulanic acid from nasal isolates (17.1%, 20/117 isolates) was higher than those from skin samples (8.3%, 16/192 isolates). Most *Staphylococcus* isolates remained sensitive to amikacin, cephalexin, amoxicillin/clavulanic acid, cefoxitin, and levofloxacin. None of the isolates were resistant to linezolid and vancomycin.

Additionally, *Staphylococcus* isolated from dogs with respiratory disease showed a higher proportion of resistance to amoxicillin, ampicillin, penicillin, ciprofloxacin, levofloxacin, erythromycin, gentamicin, and sulfamethoxazole/trimethoprim compared with those isolated from healthy dogs (*P* < 0.05). Similarly, *Staphylococcus* isolates from the skin of dogs with dermatitis demonstrated a higher proportion of resistance to penicillin compared to those from healthy dogs (S6 Table).

**Fig 3:**
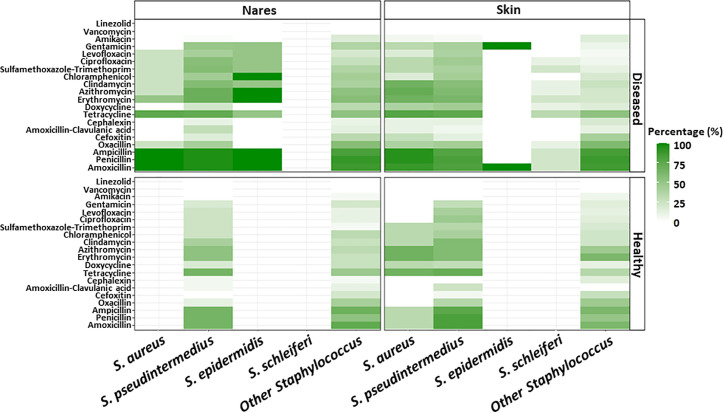
Heat map showing the percentage of *Staphylococcus* species and sample types exhibiting antibiotic resistance.

The heat map in Fig 3 illustrates the proportion of antibiotic resistance among the various *Staphylococcus* spp. In nasal samples of diseased dogs, all *S. aureus* and *S. pseudintermedius* isolates displayed a similar resistance pattern, with over 60% of isolates resistant to amoxicillin, ampicillin, penicillin, tetracycline, erythromycin, and azithromycin. Similarly, all *S. epidermidis* isolates from nasal samples were resistant to azithromycin, erythromycin, chloramphenicol, amoxicillin, ampicillin, and penicillin. In the skin samples from diseased dogs, over 88.9% of *S. aureus* isolates were resistant to amoxicillin, ampicillin, and penicillin. In healthy dogs, the proportion of resistance of isolates ranged from 0% to 69.4% from nasal samples, and 0% to 67.4% from skin samples. Notably, *S. pseudintermedius* isolates showed considerable resistance to tetracycline, penicillin, ampicillin, and amoxicillin (Fig 3).

### 3.3. Antibiotic resistance phenotypes and MDR isolates

**Fig 4:**
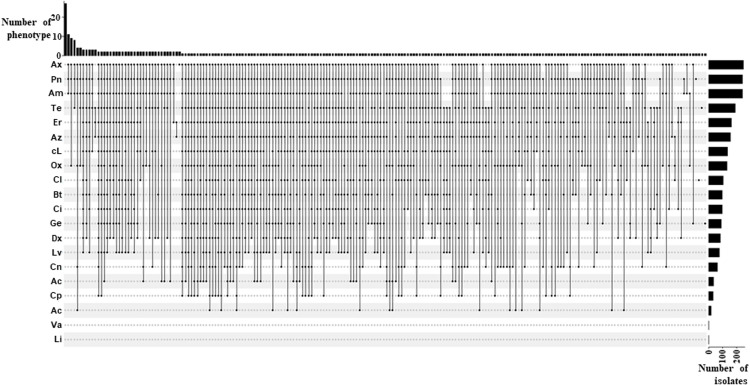
Upset plot showing the number of *Staphylococcus* spp. isolates by resistance phenotype. Black dots represent resistance to the corresponding antibiotic in the same row. The bar chart on the right shows the number of strains resistant to each antibiotic, and the bar chart above indicates the number of resistance phenotypes corresponding to the antibiotics in each column.

**Fig 5:**
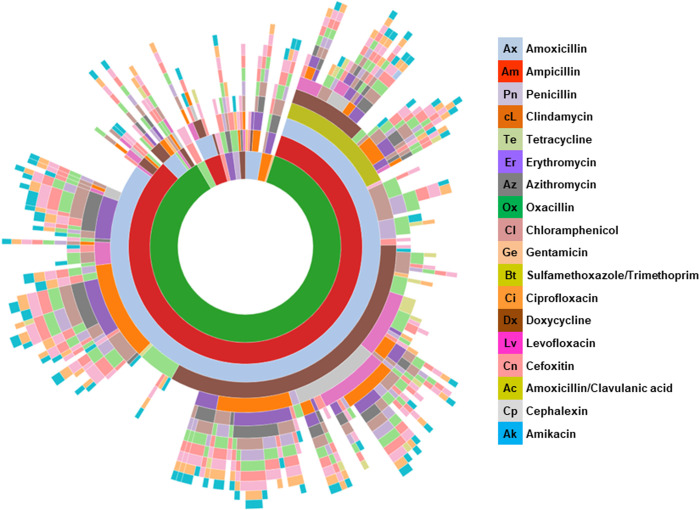
Sunburst chart illustrating the 215 antibiotic-resistance phenotypes among 309 *Staphylococcus* spp. isolates.

Fig 4 illustrates the antibiotic-resistance phenotypes of the 309 *Staphylococcus* spp. isolated. A total of 215 distinct antibiotic-resistance phenotypes were observed, with the most common being the Pn-Am-Ax (ten isolates), Pn-Am-Ax-Ox (nine isolates), and Pn-Am-Ax-Te (eight isolates). A majority of the isolates (60.5%, 187/309) exhibited MDR characteristics, defined as resistance to at least three classes of antibiotics (Fig 4 and S7 Table). Among them, 61.7% (140/227; 95% CI: 55.0–68.0%) were isolated from diseased dogs, and 57.3% (47/82, 95% CI: 45.9–68.2%) from healthy dogs. Out of the 215 antibiotic-resistance phenotypes, 85 exhibited resistance to ten or more distinct antibiotics (Fig 5). These were observed in 101 isolates belonging to different *Staphylococcus* spp., including *S. aureus* (7), *S. pseudintermedius* (71), *S. epidermidis* (1) and 22 others.

The prevalence of MDR was 68.0% (17/25) in *S*. *aureus*, 70.1% (108/154) in *S*. *pseudintermedius*, 57.1% (40/70) in other CoPS, 66.7% (2/3) in *S*. *epidermidis*, 22.2% (2/9) in *S*. *schleiferi*, and 37.5% (18/48) in other CoNS (S7 Table). For risk factor analysis, dogs sampled within 3 months of antibiotic use had a significantly higher rate of MDR *Staphylococcus* (86.7%; 95% CI: 79.2%–92.1%) compared to dogs with no antibiotic use (43.9%; 95% CI: 36.7%–51.3%), with OR = 0.12 (95% CI: 0.07–0.22; prior antibiotic use as reference). MDR prevalence was higher in *S*. *aureus* (68.0%; 95% CI: 46.5%–85.0%) compared with *S*. *schleiferi* (22.2%; 95% CI: 2.8%–60.0%), with OR = 0.13 (95% CI: 0.02–0.8; *S. aureus* as reference). It was markedly greater in *S*. *pseudintermedius* (70.1%; 95% CI: 62.2%–77.2%) compared with *S*. *schleiferi* (22.2%; 95% CI: 2.8%–60.0%) and other species (49.2%; 95% CI: 39.8%–58.5%) (S8 Table).

### 3.4. Detection of antibiotic resistance genes

The results of PCR-based detection of resistance genes in *Staphylococcus* isolates are summarized in Table 2. The genes—*aacA-aphD*, *gyrA*, and *mecA*—were more prevalent in the CoPS than in the CoNS. The most prevalent gene was *aacA-aphD* (200/309, 64.7%), identified in 172 CoPS and 28 CoNS. Additionally, 51.8% (160/309) of isolates were positive for *tetK,* including 134 CoPS and 26 CoNS. None of the CoNS isolates carried *ermA,* unlike six CoPS isolates. The *mecA* gene was present in 26.5% (82/309) of the isolates, including 73 CoPS and 9 CoNS.

**Table 2:** Distribution of antibiotic-resistance genes among *Staphylococcus* species.

Species	No. isolates	No. of isolates (%)						
		*aacA-aphD*	*tetK*	*gyrA*	*mecA*	*msrA*	*dfrA*	*ermA*
CoPS	249	172 (69.08)^a^	134 (53.82)	77 (30.92)^a^	73 (29.32)^a^	69 (27.71)	21 (8.43)	6 (2.41)
*S. aureus*	25	20	14	15	7	8	3	3
*S. pseudintermedius*	154	123	88	41	49	36	13	3
Other *Staphylococcus*	70	29	32	21	17	25	5	0
CoNS	60	28 (11.24)^b^	26 (10.44)	10 (4.02)^b^	9 (3.61)^b^	11 (4.42)	4 1.61)	0 (0)
*S. epidermidis*	3	3	2	3	2	0	2	0
*S. schleiferi*	9	3	6	0	0	0	0	0
Other *Staphylococcus*	48	22	18	7	7	11	2	0
**Total**	**309**	**200**	**160**	**87**	**82**	**80**	**25**	**6**

CoPS: Coagulase-positive *Staphylococcus*; CoNS: Coagulase-negative *Staphylococcus*.

^a,b^Values within a column with different superscript letters indicate statistically significant differences in proportions (*P* < 0.05).

**Fig 6:**
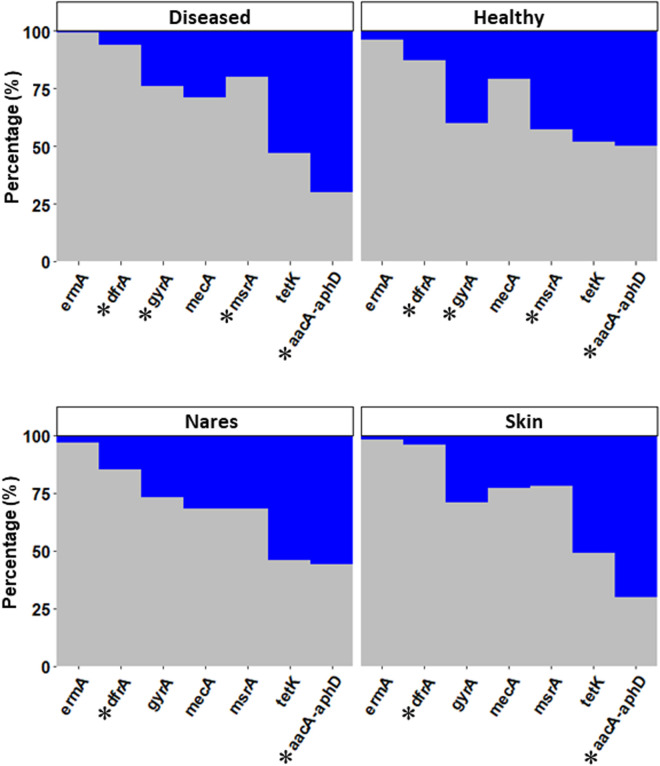
Comparison in the percentage of 309 *Staphylococcus* spp. isolates carrying resistance genes (blue) versus those not carrying resistance genes (gray), between diseased and healthy dogs (upper panel) and nares and skin samples (lower panel). (*) indicates statistically significant differences (*P* < 0.05) between groups (diseased and healthy samples, nasal and skin samples).

Among the resistance-related genes detected, *aacA-aphD* was more prevalent in *Staphylococcus* spp. isolated from diseased dogs (70.0%) compared with healthy ones (50.0%), and more common in isolates from the skin (70.3%) than from nasal samples (55.6%), with a *P *< 0.05. Conversely, resistance-associated genes such as *msrA*, *gyrA*, and *dfrA* were more common in *Staphylococcus* spp. isolated from healthy dogs (42.7%, 40.2%, and 13.4%, respectively) compared with those isolated from diseased dogs (19.8%, 23.8%, and 6.2%, respectively; *P *< 0.05). Additionally, *dfrA* was more common in the strains isolated from nasal discharge (14.5%) than those present on skin (4.2%; *P* < 0.05) (Fig 6 and S9 Table).

## 4. Discussion

This study revealed a high prevalence and species distribution of *Staphylococcus* spp. in nasal and skin samples from dogs, with a greater prevalence in diseased (78.9%) compared with healthy (56.9%) dogs (Table 1). These results are consistent with previous studies, such as those from China (60% in diseased and 41.79% in healthy dogs) [46], and Iran, where *S. pseudintermedius* was present in 50% of dogs with dermatitis compared with 40% in healthy dogs [47]. The enhanced prevalence of *Staphylococcus* spp. in diseased dogs may result from compromised immunity, which may exacerbate infection-associated conditions. Furthermore, damage to the epithelium or mucous membranes through trauma, foreign bodies, abrasions, injury to hair follicles or thermal damage, and hypersensitivity reactions alters the epithelial surface, reducing its integrity and leading to immune dysregulation or disruption of the local tissue structure. Many pathogenic *Staphylococcus* spp. possess diverse virulence factors that facilitate prolonged survival within tissues by helping evade the immune system and secreting factors that promote tissue necrosis, thereby enabling invasion of the skin and mucous membranes [48]. They also produce various toxins with superantigen properties that affect immunoregulatory and inflammatory cells, thereby exacerbating the symptoms of allergic diseases such as nasal itching, rhinorrhea, coughing, and swelling of the nasal mucosa [49]. A large proportion of *Staphylococcus* spp. isolated from the skin and mucous membranes have demonstrated a role in pyoderma [50, 51]. However, the detection rate of *Staphylococcus* spp. was not influenced by breed, gender, or location, but was markedly associated with health status, age, and management practices (S4 Table).

In this study, *S. pseudintermedius* was the most prevalent species in healthy and diseased dogs, followed by *S. aureus.* This was unsurprising, given *S. pseudintermedius* is recognized as the most common *Staphylococcus* spp. in dogs [13,46,50,51,52]. The predominance of *S. pseudintermedius* can be attributed to its presence in the normal bacterial flora, playing a significant role in bacterial diseases in dogs [4,53]. *S. aureus* was also isolated in this study, seen in diseased dogs with higher prevalence and antibiotic-resistance rates than those detected in healthy dogs. The frequent detection of *S*. *aureus* in dogs in the study is concerning, with dogs identified as potential carriers of the bacteria, posing a risk of infection or reinfection to vulnerable humans in close contact [4]. The propensity of *S. aureus* and other *Staphylococcus* spp. including *S. pseudintermedius* [12], to develop methicillin resistance and MDR, increases the potential for morbidity and mortality in both humans and dogs [26,54]. MRSA is resistant to the most commonly used antibiotics, increasing the mortality risk in infected animals. MRSA remains classified as a high-priority pathogen on the World Health Organization’s 2024 Bacterial Priority Pathogens List, and is recognized globally as one of the major causes of healthcare-associated and community-acquired infections [55–57].

The distribution of *Staphylococcus* spp. varies across studies. In Trinidad, the most common species isolated were the *S. intermedius* group (including *S. pseudintermedius*, *S. intermedius,* and *S. delphini*) [4], whereas *S. aureus, S. pseudintermedius, S. intermedius*, and more than 20 other *Staphylococcus* species have been detected in Australia [58]. However, in our study *S. intermedius* were not isolated, with the less common *S. schleiferi* detected in diseased dogs. The epidemiological understanding of *S. schleiferi* infections remains limited, as the true incidence has likely been underreported due to previous identification errors [59]. Further studies are needed to elucidate the clinical relevance of this species and its relationship with the distribution of *Staphylococcus* species seen in humans.

Antibiotic-resistant *Staphylococcus* spp. pose a substantial global challenge to human and animal health. The recent emergence of antibiotic-resistant *Staphylococcus* strains in veterinary medicine contributes to the current global health problem due to the widespread dissemination of the zoonotic superbugs. The antibiotic-resistance profile of *Staphylococcus* identified in this study revealed a complex pattern. A high prevalence of resistance (more than 75% of isolates) against several beta-lactam antibiotics, such as penicillin, ampicillin, and amoxicillin, was observed. In contrast, no resistance was detected to linezolid and vancomycin, the last resort for treating infections caused by MDR Gram-positive bacteria. This was despite linezolid-resistant CoNS being previously reported in human health settings in Vietnam [26,54]. Between 20% and 42% of study isolates were resistant to cefoxitin, and oxacillin, antibiotics used to identify MRS, with oxacillin being the antibiotic of choice for detecting MRS in several studies in Vietnam [24,26] and globally [21]. Cefoxitin, a second-generation cephalosporin, is generally not used as the first-line treatment in veterinary medicine as it is costly and primarily reserved for severe infections or those resistant to beta-lactam antibiotics. However, the increasing market availability of products containing ceftriaxone (a third-generation cephalosporin) may lead to a rise in resistance to this agent in Vietnam. Several *Staphylococcus* spp. strains in our study showed resistance to antibiotics prohibited in veterinary use such as chloramphenicol (approximately 33.3%, 103/309 isolates) and those used infrequently in veterinary medicine, such as cephalexin, cefoxitin, ciprofloxacin, levofloxacin, clindamycin, and erythromycin (S5 Table). Development of resistance in bacteria with little or no exposure to the specific antibiotic could be attributed to the vertical or horizontal transfer of antibiotic resistance-conferring genes in the environment or cross-resistance to antibiotics of the same class [60]. Furthermore, this study revealed that diseased dogs harbor more antimicrobial-resistant bacteria, which may be related to specific diseases or drugs commonly administered to diseased dogs.

In this study, the resistance to amoxicillin, ampicillin, penicillin, and tetracycline was higher in isolates from nasal samples. Beta-lactams and tetracyclines are the preferred antibiotics in Vietnamese veterinary clinics for treatment of canine respiratory diseases [61]. Antibiotic selection is often influenced by habitual practices of veterinarians, with antimicrobial susceptibility testing being infrequent. Incomplete treatment and unnecessary antibiotic use can facilitate the emergence of antibiotic-resistant *Staphylococcus* [60,62]. Our findings also highlight the risk of emergence of *Staphylococcus* superbugs and their zoonotic potential, emphasizing the need to develop appropriate antimicrobial stewardship and prevention strategies. The 2023 World Organisation for Animal Health Annual Report on antimicrobial agents intended for use in animals identified penicillins as the most commonly used class of drugs in dogs and cats across 56 nations, followed by fluoroquinolones and tetracyclines [63]. Of these, the five most reported agents were lincomycin (28%), amoxicillin (25%), doxycycline (23%), cephalexin (7%), and streptomycin (6%), which may increase the risk of resistance to these antibiotics. Other studies similarly demonstrate a wide range of *Staphylococcus* resistance to antibiotics, with beta-lactams [4,64], clindamycin, and tetracycline [51,65] being less effective against *Staphylococcus* infections in dogs [17,18,66]. In China, the resistance rates of *S*. *pseudintermedius* to azithromycin and doxycycline exceeded 80%, while those to oxacillin and sulfamethoxazole-trimethoprim were 48.6% and 64.7%, respectively [67].

MDR *Staphylococcus* isolates constituted 60.5% of the isolates (187/309). Among these, only 26.5% (82/309) were associated with the presence of *mecA*. Although methicillin resistance is of particular concern due to its ability to affect the sensitivity to all beta-lactams and its association with resistance to other antibiotic classes, resistance to different antibiotics also plays a crucial role in clinical settings. This was evidenced by our findings of *Staphylococcus* isolates displaying complex resistance to more than 15 distinct antibiotics.

The representative genes conferring resistance to beta-lactams (*mecA*), aminoglycosides (*aacA-aphD*), macrolides (*msrA, ermA*), tetracycline (*tetK*), trimethoprim (*dfrA*), and fluoroquinolones (*gyrA)* were frequently found in *S. aureus* and *S. pseudintermedius* strains; however, *ermA* was not detected in *S. epidermidis* and *S. schleiferi* strains (Table 2). It is difficult to make comparisons with the varied findings seen in previous studies on the prevalence of resistance genes due to differences in geographic and temporal designs, as well as the research populations [51,64,68,69]. Further studies are necessary to determine whether *S. pseudintermedius*, *S. epidermidis*, and *S. schleiferi* act as reservoirs of the antibiotic resistance genes and exchange them with *S. aureus* in dogs and humans.

Our analysis of antibiotic resistance-associated phenotypes and genotypes revealed that *Staphylococcus* isolates carrying the same one or two resistance-conferring genes exhibited diverse phenotypes. For example, most isolates harboring *mecA* displayed varied resistance phenotypes across multiple antibiotic classes. Furthermore, the presence or absence of a single antimicrobial resistance-conferring gene did not consistently predict susceptibility or resistance to any specific antibiotic [70]. This finding suggests that resistance-associated phenotypes can be expressed through various mechanisms, and bacteria carrying certain resistance-related genes may not always express the corresponding phenotype. These results are similar to Bertelloni et al. (2021) in Italy [51], in which 12/50 (24%) of *Staphylococcus* isolates carried *mecA*; however, only seven exhibited the related phenotypes (four *S*. *pseudintermedius*, one *S*. *aureus*, one *S*. *xylosus*, and one *S*. *chromogenes*); 15 isolates were oxacillin-resistant but lacked *mec* (seven *S*. *pseudintermedius*, five *S*. *xylosus*, one each of *S*. *simulans*, *S*. *haemolyticus*, and *S*. *capitis*); five isolates carrying *mecA* were susceptible to methicillin. A study in India reported 21 MRSA and 12 MRCoNS isolates, yet only 6/21 MRSA and 2/12 MRCoNS were positive for the *mecA* gene (genotype) [64]. The higher rate of methicillin resistance determined by phenotype when compared with the presence of *mecA* suggests that additional mechanisms are responsible for the expression of MRS phenotypes, particularly in CoNS. Our study only identified seven antibiotic resistance-associated genes, limiting our full explanation of the phenotypes based on the genotypes. While a single phenotype can be determined by multiple genes, accurate resistance profiling requires gene sequencing to clarify the discrepancies between resistance-associated phenotypes and genotypes. Further in-depth genomic studies would be able to determine the underlying mechanisms affecting the association of genotype expression with phenotype, and their linkages to other factors. Mapping antibiotic genes in different locations and study populations should be conducted to further ascertain the status of antibiotic resistance of *Staphylococcus* spp. in Vietnam, adopting a One Health approach to monitoring antimicrobial resistance.

Infections caused by opportunistic MDR pathogens constrain the choice of effective antibiotics for veterinary use. Our study shows that the carriage of MDR S*taphylococcus* in healthy dogs is common. MDR among the normal flora of clinically healthy dogs is concerning, since most had no record of prior antibiotic therapy. In this study, the higher prevalence of MDR *Staphylococcus* spp. isolated from dogs treated with antibiotics within the previous 3 months suggests that recent antibiotic administration may be a risk factor for acquiring MDR *Staphylococcus* spp.

Asymptomatic healthy dogs may also play a role in transmitting bacteria to humans. *Staphylococcus* spp. are a part of the normal bacterial flora of the skin and mucous membranes. However, under certain circumstances, the commensal bacteria can become pathogenic, causing serious infections [50,71,72,73]. Our study showed that both CoPS and CoNS were present in diseased and healthy dogs (Table 1), aligning with several previous studies [3]. While CoPS is considered to cause infections, both CoPS and CoNS have the potential to become pathogenic in animals and humans [74]. This is facilitated by a complex interplay between genetic and physiological mechanisms within the bacteria, host immune status, and environmental conditions. Under favorable conditions, commensal CoPS and CoNS can exploit opportunities to cause various infections.

Monitoring antibiotic use in veterinary clinics is essential to mitigate antibiotic-resistance in pets and to reduce the risk of zoonotic *Staphylococcus* infections in humans. More in-depth investigations are required to determine the evolution, molecular biology, and clinical impacts of MDR pathogens, particularly those with complicated antibiotic-resistance characteristics. Inappropriate antibiotic use drives selection pressure and accelerates the emergence of MDR Staphylococci. Veterinary clinics should implement practices for antimicrobial stewardship, including the use of relevant epidemiological data to guide effective antibiotic treatment choices, as well as monitoring antibiotic resistance among microbes isolated from their patients [75].

This study successfully reported the prevalence, species identification, and antibiotic resistance of *Staphylococci* in dogs in Vietnam. However, the correlation between the presence of *Staphylococci* and disease severity remains unclear. Another limitation may be the lack of molecular epidemiological analysis, which would improve the integration of our data with the findings from other studies and help better understand the global profile of canine-resident *Staphylococci*. Further investigations are necessary to uncover risk factors contributing to the prevalence of antibiotic resistance and the relationship between antibiotic-resistant *Staphylococcus* strains occurring in dogs and their owners. Further studies should elucidate the mechanism underlying antibiotic resistance, the process by which antibiotic resistance-related genotypes are converted to phenotypes, and the roles of *Staphylococcus* in causing pathogenic conditions in infected dogs. Nevertheless, it provides crucial information about *Staphylococcus* infections of veterinary importance.

## 5. Conclusions

This study details the prevalence, species distribution, and antibiotic-resistance profiles of Staphylococci isolated from nasal and skin samples of healthy and diseased dogs in Vietnam. The identified *Staphylococcus* species encompassed four species: *S. pseudintermedius* and *S. aureus* belonging to CoPS, and *S. epidermidis* and *S. schleiferi* belonging to CoNS, with *S. pseudintermedius* being the predominant. The prevalence of antibiotic-resistant *Staphylococcus* strains was substantial, exhibiting complex antibiotic resistance phenotypes. This was characterized by a notable number of MDR isolates and the presence of multiple antibiotic resistance genes. The urgent implementation of monitoring and prevention strategies for antibiotic-resistant Staphylococci in dogs and other companion animals is essential to reduce the burden and minimize potential risks to both animal and human health.

## Supporting information

S1 Fig Singleplex PCR and multiplex PCR for species identification of *Staphylococcus* isolates. (TIF)

S2 Fig Singleplex PCR and multiplex PCR for identification of antibiotic-resistance genes of *Staphylococcus* isolates. (TIF)

S1 Table Nucleotide sequences of primers used for PCR detection of *Staphylococcus* species. (DOCX)

S2 Table Nucleotide sequences of primers used for PCR detection of antibiotic-resistance genes of *Staphylococcus* species. (DOCX)

S3 Table Prevalence and species distribution of *Staphylococcus* spp. isolated from 410 healthy and diseased dogs. (DOCX)

S4 Table Risk factors associated with *Staphylococcus* spp. in dogs. (DOCX)

S5 Table Number and percentage of *Staphylococcus* isolates showing antibiotic resistance by health status and anatomical locations. (DOCX)

S6 Table Number and percentage of *Staphylococcus* isolates showing antibiotic resistance. (DOCX)

S7 Table Number of multidrug resistant *Staphylococcus* isolates per species. (DOCX)

S8 Table Risk factors associated with MDR *Staphylococcus* spp. (DOCX)

S9 Table Number and percentage of *Staphylococcus* isolates showing antibiotic-resistance genes by health status and anatomical locations. (DOCX)

S1 File Minimal data set to consist of the data required to replicate findings reported in the article. (XLSX)

## References

[1] Parte AC, Sardà Carbasse J, Meier-Kolthoff JP, Reimer LC, Göker M. List of Prokaryotic names with Standing in Nomenclature (LPSN) moves to the DSMZ. Int J Syst Evol Microbiol. 2020;70(11):5607–12. doi: 10.1099/ijsem.0.004332 32701423 PMC7723251

[2] Markey BK, Leonard FC, Archambault M, Cullinane A, Maguire D. Staphylococcus species. 2 ed. Elsevier Inc. 2013.

[3] Jung WK, Shin S, Park YK, Noh SM, Shin SR, Yoo HS, et al. Distribution and antimicrobial resistance profiles of bacterial species in stray dogs, hospital-admitted dogs, and veterinary staff in South Korea. Prev Vet Med. 2020;184:105151. doi: 10.1016/j.prevetmed.2020.105151 33011559

[4] Suepaul S, Georges K, Unakal C, Boyen F, Sookhoo J, Ashraph K, et al. Determination of the frequency, species distribution and antimicrobial resistance of Staphylococci isolated from dogs and their owners in Trinidad. PLoS One. 2021;16(7):e0254048. doi: 10.1371/journal.pone.0254048 34214140 PMC8253405

[5] Roberts E, Nuttall T, Gkekas G, Mellanby R, Fitzgerald J, Paterson G. Not just in man’s best friend: A review of *Staphylococcus pseudintermedius* host range and human zoonosis. Res Veter Sci. 2024;:105305. 38805894 10.1016/j.rvsc.2024.105305

[6] Miszczak M, Korzeniowska-Kowal A, Wzorek A, Gamian A, Szenborn L, Rypuła K. Prevalence of coagulase-negative Staphylococci (CoNS) in healthy and sick cat and dog populations in Poland. Int J Infectious Dis. 2022;116:S64–5. 10.1016/j.ijid.2021.12.152

[7] Ventrella G, Moodley A, Grandolfo E, Parisi A, Corrente M, Buonavoglia D, et al. Frequency, antimicrobial susceptibility and clonal distribution of methicillin-resistant *Staphylococcus pseudintermedius* in canine clinical samples submitted to a veterinary diagnostic laboratory in Italy: A 3-year retrospective investigation. Vet Microbiol. 2017;211:103–6. doi: 10.1016/j.vetmic.2017.09.015 29102103

[8] Qekwana DN, Naidoo V, Oguttu JW, Odoi A. Occurrence and predictors of bacterial respiratory tract infections and antimicrobial resistance among isolates from dogs presented with lower respiratory tract infections at a referral veterinary hospital in South Africa. Front Vet Sci. 2020;7:304. doi: 10.3389/fvets.2020.00304 32582780 PMC7280450

[9] An NV, Hoang LH, Le HHL, Thai SN, Hong LT, Viet TT, et al. Distribution and antibiotic resistance characteristics of bacteria isolated from blood culture in a teaching hospital in Vietnam during 2014–2021. Infect Drug Resistance. 2023:1677–92. 10.2147/idr.s402278 36992965 PMC10041986

[10] Nemeghaire S, Argudín MA, Feßler AT, Hauschild T, Schwarz S, Butaye P. The ecological importance of the *Staphylococcus sciuri* species group as a reservoir for resistance and virulence genes. Vet Microbiol. 2014;171(3–4):342–56. doi: 10.1016/j.vetmic.2014.02.005 24629775

[11] Lakhundi S, Zhang K. Methicillin-resistant *Staphylococcus aureus*: Molecular characterization, evolution, and epidemiology. Clin Microbiol Rev. 2018;31(4):e00020-18. doi: 10.1128/CMR.00020-18 30209034 PMC6148192

[12] Phumthanakorn N, Fungwithaya P, Chanchaithong P, Prapasarakul N. Enterotoxin gene profile of methicillin-resistant *Staphylococcus pseudintermedius* isolates from dogs, humans and the environment. J Med Microbiol. 2018;67(6):866–73. doi: 10.1099/jmm.0.000748 29724270

[13] Burke M, Santoro D. Prevalence of multidrug-resistant coagulase-positive staphylococci in canine and feline dermatological patients over a 10-year period: a retrospective study. Microbiology. 2023;169(2). 10.1099/mic.0.001300 36786549 PMC10197874

[14] de Jong A, Youala M, El Garch F, Simjee S, Rose M, Morrissey I, et al. Antimicrobial susceptibility monitoring of canine and feline skin and ear pathogens isolated from European veterinary clinics: results of the ComPath Surveillance programme. Vet Dermatol. 2020;31(6):431-e114. doi: 10.1111/vde.12886 32924232

[15] Gómez-Beltrán DA, Villar D, López-Osorio S, Ferguson D, Monsalve LK, Chaparro-Gutiérrez JJ. Prevalence of antimicrobial resistance in bacterial isolates from dogs and cats in a veterinary diagnostic laboratory in Colombia from 2016-2019. Vet Sci. 2020;7(4):173. doi: 10.3390/vetsci7040173 33182667 PMC7712406

[16] de Menezes MP, Facin AC, Cardozo MV, Costa MT, Moraes PC. Evaluation of the resistance profile of bacteria obtained from infected sites of dogs in a veterinary teaching hospital in Brazil: A retrospective study. Top Companion Anim Med. 2021;42:100489. doi: 10.1016/j.tcam.2020.100489 33144265

[17] Cuny C, Layer-Nicolaou F, Weber R, Köck R, Witte W. Colonization of dogs and their owners with *Staphylococcus aureus* and *Staphylococcus pseudintermedius* in households, veterinary practices, and healthcare facilities. Microorganisms. 2022;10(4):677. doi: 10.3390/microorganisms10040677 35456729 PMC9024920

[18] Jin M, Osman M, Green BA, Yang Y, Ahuja A, Lu Z, et al. Evidence for the transmission of antimicrobial resistant bacteria between humans and companion animals: A scoping review. One Health. 2023;17:100593. doi: 10.1016/j.onehlt.2023.100593 37448771 PMC10336692

[19] Cengiz S, Okur S, Oz C, Turgut F, Gumurcinler B, Sevuk NS, et al. Prevalence and clonal diversity of methicillin-resistant *Staphylococcus aureus* and methicillin-resistant *Staphylococcus pseudintermedius* isolated from dogs and cats with eye discharge. Acta microbiologica et immunologica Hungarica. 2023;70(2):134–41. 10.1556/030.2023.01899 36723933

[20] Viegas FM, Santana JA, Silva BA, Xavier RGC, Bonisson CT, Câmara JLS, et al. Occurrence and characterization of methicillin-resistant *Staphylococcus* spp. in diseased dogs in Brazil. PLoS One. 2022;17(6):e0269422. doi: 10.1371/journal.pone.0269422 35657980 PMC9165789

[21] Moses IB, Santos FF, Gales AC. Human Colonization and Infection by *Staphylococcus pseudintermedius*: An Emerging and Underestimated Zoonotic Pathogen. Microorganisms. 2023;11(3):581. doi: 10.3390/microorganisms11030581 36985155 PMC10057476

[22] Ho CS, Wong CTH, Aung TT, Lakshminarayanan R, Mehta JS, Rauz S, et al. Antimicrobial resistance: a concise update. Lancet Microbe. 2025;6(1):100947. doi: 10.1016/j.lanmic.2024.07.010 39305919

[23] Nguyen Thai S, Vu Thi Thu H, Vu Thi Kim L, Do Thi Quynh N, Tran Thi Hai A, Tang Thi N, et al. First report on multidrug-resistant methicillin-resistant *Staphylococcus aureus* isolates in children admitted to tertiary hospitals in Vietnam. J Microbiol Biotechnol. 2019;29(9):1460–9. doi: 10.4014/jmb.1904.04052 31434169

[24] Tran KQ, Nguyen TTD, Pham VH, Pham QM, Tran HD. Pathogenic role and antibiotic resistance of methicillin-resistant *Staphylococcus aureus* (MRSA) strains causing severe community-acquired pneumonia in vietnamese children. Adv Respir Med. 2023;91(2):135–45. doi: 10.3390/arm91020012 37102779 PMC10135923

[25] Thai Son N, Thu Huong VT, Kim Lien VT, Quynh Nga DT, Hai Au TT, Thu Hang PT, et al. Antimicrobial resistance profile and molecular characteristics of *Staphylococcus aureus* isolates from hospitalized adults in three regions of vietnam. Jpn J Infect Dis. 2020;73(3):193–200. doi: 10.7883/yoken.JJID.2019.239 31875603

[26] Nguyen LTT, Nguyen KNT, Le PNTA, Cafini F, Pascoe B, Sheppard SK, et al. The emergence of plasmid-borne cfr-mediated linezolid resistant-staphylococci in Vietnam. J Glob Antimicrob Resist. 2020;22:462–5. doi: 10.1016/j.jgar.2020.04.008 32348904

[27] Chao NV, Dung HT, Thanh Tam VT, Hang PT, Hien BT. The role of veterinary drug use in driving antimicrobial resistance of *Staphylococcus aureus* isolates in smallholder swine farms in Central Vietnam. Open Vet J. 2025;15(2):847–62. doi: 10.5455/OVJ.2025.v15.i2.34 40201839 PMC11974315

[28] Son HM, Duc HM. Prevalence and phage-based biocontrol of methicillin-resistant *Staphylococcus aureus* isolated from raw milk of cows with subclinical mastitis in vietnam. Antibiotics (Basel). 2024;13(7):638. doi: 10.3390/antibiotics13070638 39061320 PMC11273874

[29] Cochran WG. Sampling techniques. John Wiley & Sons. 1977.

[30] Hillier A, Lloyd DH, Weese JS, Blondeau JM, Boothe D, Breitschwerdt E, et al. Guidelines for the diagnosis and antimicrobial therapy of canine superficial bacterial folliculitis (Antimicrobial Guidelines Working Group of the International Society for Companion Animal Infectious Diseases). Vet Dermatol. 2014;25(3):163-e43. doi: 10.1111/vde.12118 24720433

[31] Hassanzadeh S, Pourmand MR, Afshar D, Dehbashi S, Mashhadi R. TENT: A rapid DNA extraction method of *Staphylococcus aureus*. Iran J Public Health. 2016;45(8):1093–5. 27928541 PMC5139972

[32] Weisburg WG, Barns SM, Pelletier DA, Lane DJ. 16S ribosomal DNA amplification for phylogenetic study. J Bacteriol. 1991;173(2):697–703. doi: 10.1128/jb.173.2.697-703.1991 1987160 PMC207061

[33] Sasaki T, Tsubakishita S, Tanaka Y, Sakusabe A, Ohtsuka M, Hirotaki S, et al. Multiplex-PCR method for species identification of coagulase-positive Staphylococci. J Clin Microbiol. 2010;48(3):765–9. doi: 10.1128/JCM.01232-09 20053855 PMC2832457

[34] Morar A, Ban-Cucerzan A, Herman V, Tîrziu E, Sallam KI, Abd-Elghany SM, et al. Multidrug resistant coagulase-positive *staphylococcus aureus* and their enterotoxins detection in traditional cheeses marketed in Banat Region, Romania. Antibiotics (Basel). 2021;10(12):1458. doi: 10.3390/antibiotics10121458 34943670 PMC8698683

[35] Mason WJ, Blevins JS, Beenken K, Wibowo N, Ojha N, Smeltzer MS. Multiplex PCR protocol for the diagnosis of staphylococcal infection. J Clin Microbiol. 2001;39(9):3332–8. doi: 10.1128/JCM.39.9.3332-3338.2001 11526172 PMC88340

[36] Hirotaki S, Sasaki T, Kuwahara-Arai K, Hiramatsu K. Rapid and accurate identification of human-associated Staphylococci by use of multiplex PCR. J Clin Microbiol. 2011;49(10):3627–31. doi: 10.1128/JCM.00488-11 21832022 PMC3187289

[37] CLSI. Performance standards for antimicrobial susceptibility testing. 100. Wayne, PA: Clinical and Laboratory Standards Institute. 2020.

[38] Magiorakos A-P, Srinivasan A, Carey RB, Carmeli Y, Falagas ME, Giske CG, et al. Multidrug-resistant, extensively drug-resistant and pandrug-resistant bacteria: an international expert proposal for interim standard definitions for acquired resistance. Clin Microbiol Infect. 2012;18(3):268–81. doi: 10.1111/j.1469-0691.2011.03570.x 21793988

[39] Wheat W, Simiyu B, Andonie G, Bellfi L. Clinical impact of vancomycin MIC on outcomes in patients with coagulase-negative Staphylococcal bacteremia. Clinical therapeutics. 2024. 38493003 10.1016/j.clinthera.2024.01.012

[40] Meshref A, Omer M. Detection of (mecA) gene in methicillin resistant *Staphylococcus aureus* (MRSA) at Prince A/Rhman Sidery. J Medical Genet Genomics. 2011;3:41–5.

[41] Strommenger B, Kettlitz C, Werner G, Witte W. Multiplex PCR assay for simultaneous detection of nine clinically relevant antibiotic resistance genes in *Staphylococcus aureus*. J Clin Microbiol. 2003;41(9):4089–94. doi: 10.1128/JCM.41.9.4089-4094.2003 12958230 PMC193808

[42] Timsina R, Shrestha U, Singh A, Timalsina B. Inducible clindamycin resistance and erm genes in *Staphylococcus aureus* in school children in Kathmandu, Nepal. Future Science OA. 2021;7(1):FSO361. 10.2144/fsoa-2020-0092 PMC7787115 33437500

[43] Lina G, Quaglia A, Reverdy ME, Leclercq R, Vandenesch F, Etienne J. Distribution of genes encoding resistance to macrolides, lincosamides, and streptogramins among Staphylococci. Antimicrob Agents Chemother. 1999;43(5):1062–6. doi: 10.1128/AAC.43.5.1062 10223914 PMC89111

[44] Shittu AO, Okon K, Adesida S, Oyedara O, Witte W, Strommenger B, et al. Antibiotic resistance and molecular epidemiology of *Staphylococcus aureus* in Nigeria. BMC Microbiol. 2011;11:92. doi: 10.1186/1471-2180-11-92 21545717 PMC3112067

[45] Dubin DT, Fitzgibbon JE, Nahvi MD, John JF. Topoisomerase sequences of coagulase-negative staphylococcal isolates resistant to ciprofloxacin or trovafloxacin. Antimicrob Agents Chemother. 1999;43(7):1631–7. doi: 10.1128/AAC.43.7.1631 10390214 PMC89335

[46] Wang Z, Guo L, Li J, Li J, Cui L, Dong J, et al. Antibiotic resistance, biofilm formation, and virulence factors of isolates of *staphylococcus pseudintermedius* from healthy dogs and dogs with keratitis. Front Vet Sci. 2022;9:903633. doi: 10.3389/fvets.2022.903633 36032292 PMC9399793

[47] Naziri Z, Majlesi M. Comparison of the prevalence, antibiotic resistance patterns, and biofilm formation ability of methicillin-resistant *Staphylococcus pseudintermedius* in healthy dogs and dogs with skin infections. Vet Res Commun. 2023;47(2):713–21. doi: 10.1007/s11259-022-10032-7 36327008

[48] Loy JD. Staphylococcus: Clinical significance in animals. Clinical Veterinary Microbiology. 2013.

[49] Janeczek K, Emeryk A, Zimmer Ł, Poleszak E, Ordak M. Nasal carriage of *Staphylococcus aureus* in children with grass pollen-induced allergic rhinitis and the effect of polyvalent mechanical bacterial lysate immunostimulation on carriage status: A randomized controlled trial. Immun Inflamm Dis. 2022;10(3):e584. doi: 10.1002/iid3.584 34965026 PMC8926494

[50] Lord J, Millis N, Jones RD, Johnson B, Kania SA, Odoi A. Patterns of antimicrobial, multidrug and methicillin resistance among *Staphylococcus* spp. isolated from canine specimens submitted to a diagnostic laboratory in Tennessee, USA: a descriptive study. BMC Vet Res. 2022;18(1):91. doi: 10.1186/s12917-022-03185-9 35255907 PMC8903740

[51] Bertelloni F, Cagnoli G, Ebani VV. Virulence and Antimicrobial Resistance in Canine *Staphylococcus* spp. Isolates. Microorganisms. 2021;9(3):515. doi: 10.3390/microorganisms9030515 33801518 PMC7998746

[52] Nakaminami H, Okamura Y, Tanaka S, Wajima T, Murayama N, Noguchi N. Prevalence of antimicrobial-resistant Staphylococci in nares and affected sites of pet dogs with superficial pyoderma. J Vet Med Sci. 2021;83(2):214–9. doi: 10.1292/jvms.20-0439 33342967 PMC7972875

[53] Elshabrawy MA, Abouelhag HA, Khairy EA, Marie HS, Hakim AS. Molecular divergence of *Staphylococcus aureus* isolated from dogs and cats. Jordan J Biological Sci. 2020;13(2).

[54] An NV, Hai LHL, Luong VH, Vinh NTH, Hoa PQ, Hung LV, et al. Antimicrobial resistance patterns of *Staphylococcus aureus* isolated at a general hospital in vietnam between 2014 and 2021. Infect Drug Resist. 2024;17:259–73. doi: 10.2147/IDR.S437920 38283112 PMC10822110

[55] European Antimicrobial Resistance Collaborators. The burden of bacterial antimicrobial resistance in the WHO European region in 2019: a cross-country systematic analysis. Lancet Public Health. 2022;7(11):e897–913. doi: 10.1016/S2468-2667(22)00225-0 36244350 PMC9630253

[56] Antimicrobial Resistance Collaborators. Global burden of bacterial antimicrobial resistance in 2019: a systematic analysis. Lancet. 2022;399(10325):629–55. doi: 10.1016/S0140-6736(21)02724-0 35065702 PMC8841637

[57] World Health Organization. WHO bacterial priority pathogens list, 2024: bacterial pathogens of public health importance, to guide research, development, and strategies to prevent and control antimicrobial resistance. World Health Organization. 2024.

[58] Ma GC, Worthing KA, Ward MP, Norris JM. Commensal Staphylococci including methicillin-resistant *Staphylococcus aureus* from dogs and cats in remote new south wales, australia. Microb Ecol. 2020;79(1):164–74. doi: 10.1007/s00248-019-01382-y 31049616

[59] Naing SY, Duim B, Broens EM, Schweitzer V, Zomer A, van der Graaf-van Bloois L, et al. Molecular characterization and clinical relevance of taxonomic reassignment of *Staphylococcus schleiferi* subspecies into two separate species, *Staphylococcus schleiferi* and *Staphylococcus coagulans*. Microbiol Spectr. 2023;11(2):e0467022. doi: 10.1128/spectrum.04670-22 36853031 PMC10101015

[60] Fymat AL. Antibiotics and antibiotic resistance. Biomedical J Sci Technical Res. 2017;1(1):1–16.

[61] Loan NVTH, Nguyen NPT, Nguyen KTP, Tran TNT, Cuc DTK, Anh NTL. Using antibiotics for respiratory disease treatment in dogs of Ho Chi Minh City. Animal Husbandry Associat Vietnam. 2024;297:73–80.

[62] Rahman MM, Alam Tumpa MA, Zehravi M, Sarker MT, Yamin M, Islam MR, et al. An overview of antimicrobial stewardship optimization: The use of antibiotics in humans and animals to prevent resistance. Antibiotics (Basel). 2022;11(5):667. doi: 10.3390/antibiotics11050667 35625311 PMC9137991

[63] WOAH. Annual report on antimicrobial agents intended for use in animals. World Organisation for Animal Health. 2023.

[64] Chaudhari SS, Chauhan HC, Sharma KK, Patel SS, Patel AC, Mohapatra SK, et al. Antibiotic susceptibility pattern of canine coagulase positive and coagulase negative *Staphylococcus* spp. in a hot and dry region of india. Top Companion Anim Med. 2022;50:100679. doi: 10.1016/j.tcam.2022.100679 35688355

[65] Jantorn P, Heemmamad H, Soimala T, Indoung S, Saising J, Chokpaisarn J, et al. Antibiotic resistance profile and biofilm production of *Staphylococcus pseudintermedius* isolated from dogs in thailand. Pharmaceuticals (Basel). 2021;14(6):592. doi: 10.3390/ph14060592 34203050 PMC8234208

[66] Kjellman EE, Slettemeås JS, Small H, Sunde M. Methicillin-resistant *Staphylococcus pseudintermedius* (MRSP) from healthy dogs in Norway - occurrence, genotypes and comparison to clinical MRSP. Microbiologyopen. 2015;4(6):857–66. doi: 10.1002/mbo3.258 PMC4694142 26423808

[67] Wang Q, Chen S, Ma S, Jiao Y, Hong H, Wang S, et al. Antimicrobial resistance and risk factors of canine bacterial skin infections. Pathogens. 2025;14(4):309. doi: 10.3390/pathogens14040309 40333053 PMC12030357

[68] Bruce SA, Smith JT, Mydosh JL, Ball J, Needle DB, Gibson R, et al. Shared antibiotic resistance and virulence genes in *Staphylococcus aureus* from diverse animal hosts. Sci Rep. 2022;12(1):4413. doi: 10.1038/s41598-022-08230-z 35292708 PMC8924228

[69] Ferrer L, García-Fonticoba R, Pérez D, Viñes J, Fàbregas N, Madroñero S, et al. Whole genome sequencing and de novo assembly of *Staphylococcus pseudintermedius*: a pangenome approach to unravelling pathogenesis of canine pyoderma. Vet Dermatol. 2021;32(6):654–63. doi: 10.1111/vde.13040 34796561

[70] Rasheed H, Ijaz M, Ahmed A, Javed MU, Shah SFA, Anwaar F. Discrepancies between phenotypic and genotypic identification methods of antibiotic resistant genes harboring *Staphylococcus aureus*. Microb Pathog. 2023;184:106342. doi: 10.1016/j.micpath.2023.106342 37704062

[71] Burke M, Santoro D. Prevalence of multidrug-resistant coagulase-positive Staphylococci in canine and feline dermatological patients over a 10-year period: a retrospective study. Microbiology (Reading). 2023;169(2):001300. doi: 10.1099/mic.0.001300 36786549 PMC10197874

[72] Kawakami T, Shibata S, Murayama N, Nagata M, Nishifuji K, Iwasaki T, et al. Antimicrobial susceptibility and methicillin resistance in *Staphylococcus pseudintermedius* and *Staphylococcus schleiferi* subsp. coagulans isolated from dogs with pyoderma in Japan. J Vet Med Sci. 2010;72(12):1615–9. doi: 10.1292/jvms.10-0172 20703027

[73] Hauschild T, Wójcik A. Species distribution and properties of Staphylococci from canine dermatitis. Res Vet Sci. 2007;82(1):1–6. doi: 10.1016/j.rvsc.2006.04.004 17126372

[74] Park S, Ronholm J. *Staphylococcus aureus* in agriculture: lessons in evolution from a multispecies pathogen. Clin Microbiol Rev. 2021;34(2):e00182-20. doi: 10.1128/CMR.00182-20 33568553 PMC7950364

[75] Caneschi A, Bardhi A, Barbarossa A, Zaghini A. The use of antibiotics and antimicrobial resistance in veterinary medicine, a complex phenomenon: a narrative review. Antibiotics (Basel). 2023;12(3):487. doi: 10.3390/antibiotics12030487 36978354 PMC10044628

