# Supplementary figures and images for: Prevalence, species identification, and antibiotic resistance of Staphylococci in dogs visiting veterinary clinics in Vietnam
Source: PLoS One. 2025 Jul 24;20(7):e0328472. doi: 10.1371/journal.pone.0328472 (PMC12289047; doi:10.1371/journal.pone.0328472)

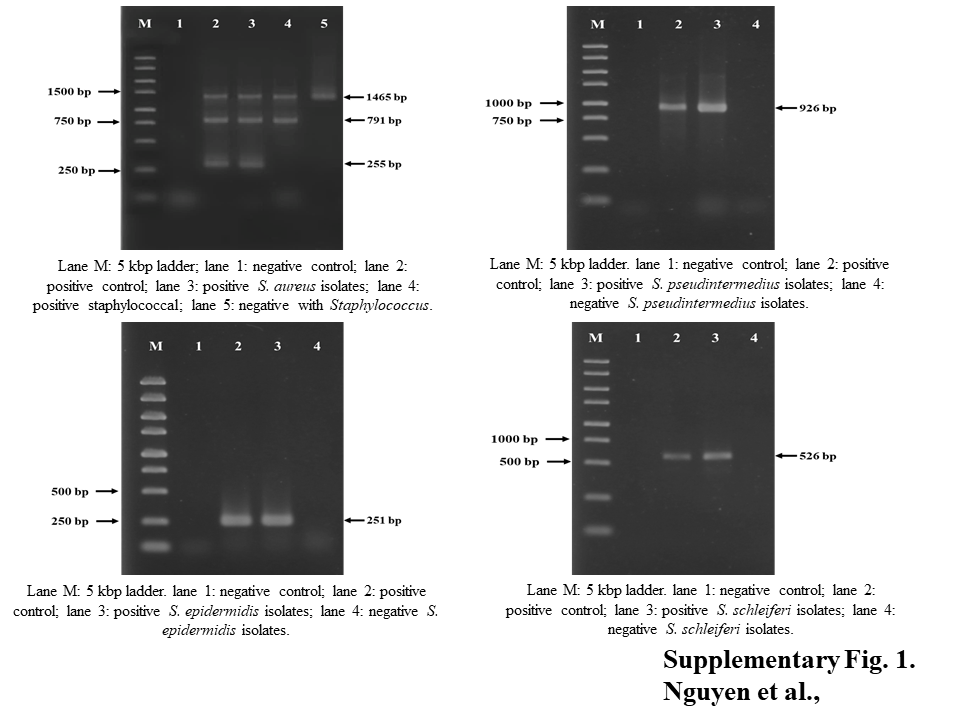

Supplement: S1 Fig — (TIF) [file pone.0328472.s001.tif]

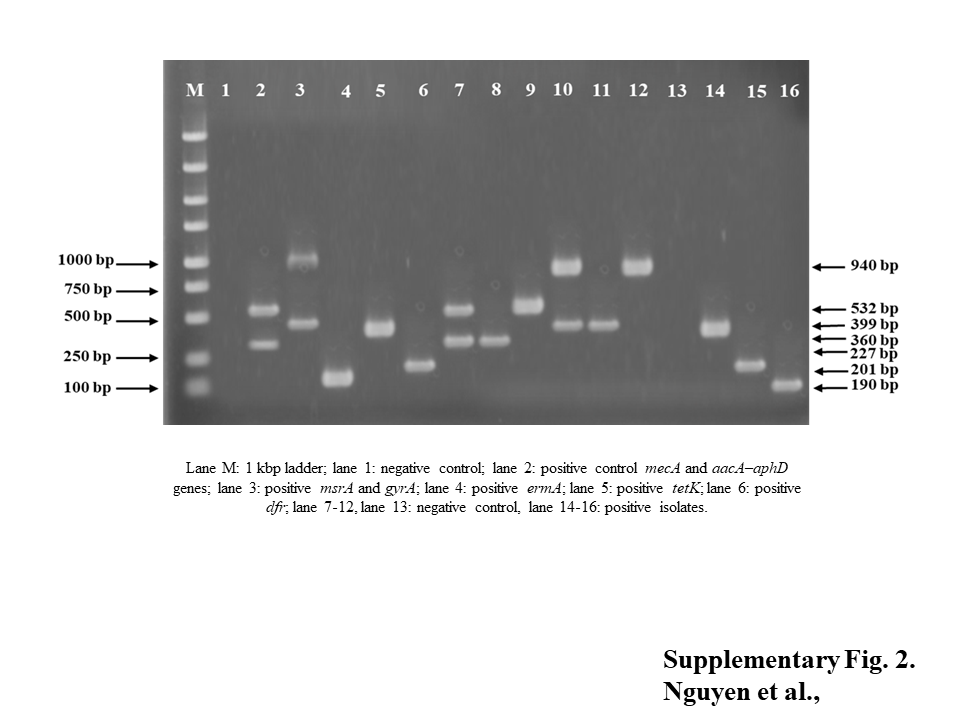

Supplement: S2 Fig — (TIF) [file pone.0328472.s002.tif]
