# Supplementary material for: Prevalence, species identification, and antibiotic resistance of Staphylococci in dogs visiting veterinary clinics in Vietnam
Source: PLoS One. 2025 Jul 24;20(7):e0328472. doi: 10.1371/journal.pone.0328472 (PMC12289047; doi:10.1371/journal.pone.0328472)
Supplement: S1 Table — (DOCX) [file pone.0328472.s003.docx]

**S1 Table.**

Nucleotide sequences of primers used for PCR detection of *Staphylococcus* species.

| **Target gene** | **Primer** | **Sequence (5’ – 3’)** | **Amplicon**  **size (bp)** | **Annealing**  **T_m_ (^o^C)** | **Reference** |
| --- | --- | --- | --- | --- | --- |
| *16S rRNA* | 27F  1492R | CAGAGTTTGATCCTGGCT  AGGAGGTGATCCAGCCGCA | 1,465 | 55 | Weisburg et al. (1991) |
| *16s RNA Staphylococcus* | Nuc-alF1  Nuc-alR | CCTATAAGACTGGGATAACTTCGGG  CTTTGAGTTTCAACCTTGCGGTCG | 791 | 52 | Mason et al. (2001) |
| *nuc*  *(S. epidermidis)* | epi-F  epi-R | TTGTAAACCATTCTGGACCG  ATGCGTGAGATACTTCTTCG | 251 | 58 | Hirotaki et al. (2011) |
| *nuc*  *(S. aureus)* | nuc-1  nuc-2 | TCAGCAAATGCATCACAAACAG  CGTAAATGCACTTGCTTCAGG | 255 | 55 | Morar et al. (2021) |
| *nuc*  *(S. pseudintermedius)* | pse-F2  pse-R5 | TRGGCAGTAGGATTCGTTAA  CTTTTGTGCTYCMTTTTGG | 926 | 57 | Sasaki et al. (2010) |
| *nuc*  *(S. intermedius)* | in-F  in-R3 | CATGTCATATTATTGCGAA  TGA  AGGACCATCACCATTGACA  TATTGAAACC | 430 | 56 | Sasaki et al. (2010) |
| *S. schleiferi* | sch-F  sch-R | AATGGCTACAATGATAATCACTAA  CATATCTGTCTTTCGGCGCG | 526 | 56 | Sasaki et al. (2010) |
