# Supplementary material for: Prevalence, species identification, and antibiotic resistance of Staphylococci in dogs visiting veterinary clinics in Vietnam
Source: PLoS One. 2025 Jul 24;20(7):e0328472. doi: 10.1371/journal.pone.0328472 (PMC12289047; doi:10.1371/journal.pone.0328472)
Supplement: S2 Table — (DOCX) [file pone.0328472.s004.docx]

**S2 Table.**

Nucleotide sequences of primers used for PCR detection of antibiotic-resistance genes of *Staphylococcus* species.

| **Antibiotic class** | **Gene** | **Sequence (5’ – 3’)** | **Amplicon**  **size (bp)** | **Annealing**  **T_m_ (^o^C)** | **Reference** |
| --- | --- | --- | --- | --- | --- |
| Beta-lactam | *mecA* | AAAATCGATGGTAAAGGTTGGC | 532 | 55^o^C | Meshref and Omer (2011) |
|  |  | AGTTCTGCAGTACCGGATTTGC |  |  |  |
| Aminoglycoside | *aacA-aphD* | TAATCCAAG AGCAATAAGGGC | 227 | 55^o^C | Strommenger et al. (2003) |
|  |  | GCCACACTATCATAACCACTA |  |  |  |
| Macrolide, lincosamide, and streptogramin B | *ermA* | AAGCGGTAAACCCCTCTGA | 190 | 55^o^C | Timsina et al. (2021) |
|  |  | TTCGCAAATCCCTTCTCAAC |  |  |  |
| Tetracycline | *tetK* | GTAGCGACAATAGGTAATAGT | 360 | 48^o^C | Strommenger et al. (2003) |
|  |  | GTAGTGACAATAAACCTCCTA |  |  |  |
| Macrolide | *msrA* | GGCACAATAAGAGTGTTTAA AGG | 940 | 50^o^C | Lina et al. (1999) |
|  |  | AAGTTATATCATGAATAGAT TGTCCTGTT |  |  |  |
| Trimethoprim | *dfrA* | CTCACGATAAACAAAGAGTCA | 201 | 50^o^C | Shittu et al. (2011) |
|  |  | CAATCATTG CTTCGTATAACG |  |  |  |
| Fluoroquinolones | *gyrA* | ATGGCTGAATTACCTCAATC  CATCATAGTTATCGATGAAATC | 399 | 55^o^C | Dubin et al. (1999) |
