# Supplementary material for: Prevalence, species identification, and antibiotic resistance of Staphylococci in dogs visiting veterinary clinics in Vietnam
Source: PLoS One. 2025 Jul 24;20(7):e0328472. doi: 10.1371/journal.pone.0328472 (PMC12289047; doi:10.1371/journal.pone.0328472)
Supplement: S3 Table — (DOCX) [file pone.0328472.s005.docx]

# S3 Table.

# Prevalence and species distribution of *Staphylococcus* spp. isolated from 410 healthy and diseased dogs.

| **Sample type** | **No.**  **samples** | **No. of**  **positive**  **samples** | **Percentage (%)**  **(95% CI)** | **OR**  **(95% CI)** |  | **No.**  **isolates** | **No. (%) of CoPS** | | | |  | **No. (%) of CoNS** | | | |
| --- | --- | --- | --- | --- | --- | --- | --- | --- | --- | --- | --- | --- | --- | --- | --- |
|  |  |  |  |  |  |  | **n**  **(%)** | ***S.***  ***aureus*** | ***S.***  ***pseudintermedius*** | **Others** |  | **n**  **(%)** | ***S.***  ***epidermidis*** | ***S.***  ***schleiferi*** | **Others** |
| **Health status** | | | | | | | | | | | | | | | |
| Healthy | 144 | 82 | 56.9  (48.4–65.2) | Reference |  | 82 | 63  (76.8) | 3  (3.7) | 34  (41.5) | 26  (31.7) |  | 19  (23.2) | 0  (0) | 0  (0) | 19  (23.2) |
| Diseased | 266 | 210 | 78.9  (73.6–83.7) | 2.84  (1.82–4.41) |  | 227 | 186  (81.9) | 22  (9.7) | 120  (52.9) | 44  (19.4) |  | 41  (18.1) | 3  (1.3) | 9  (3.9) | 29  (12.8) |
| **Total** | **410** | **292** | **71.2**  **(66.6–75.6)** |  |  | **309** | **249**  **(80.6)** | **25**  **(8.1)** | **154**  **(49.8)** | **70**  **(22.7)** |  | **60**  **(19.4)** | **3**  **(0.9)** | **9**  **(2.9)** | **48**  **(15.5)** |
| **Anatomical location** | | | | | | | | | | | | | | | |
| Nares | 184 | 111 | 60.3  (52.9–67.5) | Reference |  | 117 | 94  (80.3) | 4  (3.4) | 62  (53.0) | 28  (23.9) |  | 23  (19.7) | 2  (1.7) | 0  (0) | 21  (18.0) |
| Skin | 226 | 181 | 80.1  (74.3–85.1) | 2.65  (1.70–4.11) |  | 192 | 155  (80.7) | 21  (10.9) | 92  (47.9) | 42  (21.9) |  | 37  (19.3) | 1  (0.5) | 9  (4.7) | 27  (14.1) |
| **Total** | **410** | **292** | **71.2**  **(66.6–75.6)** |  |  | **309** | **249**  **(80.6)** | **25**  **(8.1)** | **154**  **(49.8)** | **70**  **(22.7)** |  | **60**  **(19.4)** | **3**  **(0.9)** | **9**  **(2.9)** | **48**  **(15.5)** |
