# Supplementary material for: Prevalence, species identification, and antibiotic resistance of Staphylococci in dogs visiting veterinary clinics in Vietnam
Source: PLoS One. 2025 Jul 24;20(7):e0328472. doi: 10.1371/journal.pone.0328472 (PMC12289047; doi:10.1371/journal.pone.0328472)
Supplement: S4 Table — (DOCX) [file pone.0328472.s006.docx]

# S4 Table. Risk factors associated with *Staphylococcus* spp. in dogs.

| **Factors** | | **Nares** | | | |  | **Skin** | | | |
| --- | --- | --- | --- | --- | --- | --- | --- | --- | --- | --- |
|  |  | **No. of samples** | **No. of positive samples** | **Percentage (%)**  **(95% CI)** | **OR**  **(95% CI)** |  | **No. of sample** | **No. of positive sample** | **Percentage (%)**  **(95% CI)** | **OR**  **(95% CI)** |
| Health status | Diseased | 112 | 75 | 67.0  (57.4–75.6) | Reference  0.49 (0.27–0.91) |  | 154 | 135 | 87.7  (81.4–92.4) | Reference  0.25 (0.13–0.49) |
|  | Healthy | 72 | 36 | 50.0  (38.0–62.0) |  |  | 72 | 46 | 63.9  (51.7–74.9) |  |
| Breed | Foreign | 131 | 85 | 64.9  (56.0–73.0) | Reference  0.52 (0.27–1.00) |  | 152 | 124 | 81.6  (74.5–87.4) | Reference  0.76 (0.38–1.49) |
|  | Domestic | 53 | 26 | 49.1  (35.1–63.2) |  |  | 74 | 57 | 77.0  (65.8–86.0) |  |
| Gender | Male | 90 | 58 | 64.4  (53.7–74.3) | Reference  0.71 (0.39–1.29) |  | 112 | 92 | 82.1  (73.8–88.7) | Reference  0.77 (0.40–1.49) |
|  | Female | 94 | 53 | 56.4  (45.8–66.6) |  |  | 114 | 89 | 78.1  (69.4–85.3) |  |
| Age (years) | < 1 | 59 | 22 | 37.3  (25.0–50.9) | Reference  2.38 (1.13–5.01)  7.71 (3.40–17.46) |  | 46 | 24 | 52.2  (37.0–67.1) | Reference  4.45 (2.11–9.38)  25.21 (5.49–115.82) |
|  | 1–5 | 58 | 34 | 58.6  (44.9–71.4) |  |  | 123 | 102 | 82.9  (75.1–89.1) |  |
|  | > 5 | 67 | 55 | 82.1  (70.8–90.4) |  |  | 57 | 55 | 96.5  (87.9–99.6) |  |
| Management practices | Free-ranging | 66 | 31 | 47.0  (34.6–59.7) | Reference  1.68 (0.86–3.25)  5.48 (2.13–14.13) |  | 41 | 39 | 95.1  (83.5–99.4) | Reference  0.22 (0.05–0.99)  0.12 (0.03–0.56) |
|  | Semi-confined | 77 | 46 | 59.7  (47.9–70.8) |  |  | 106 | 86 | 81.1  (72.4–88.1) |  |
|  | Confined | 41 | 34 | 82.9  (67.9–92.9) |  |  | 79 | 56 | 70.9  (59.6–80.6) |  |
| Location | Urban | 129 | 83 | 64.3  (55.4–72.6) | Reference  0.57 (0.30–1.09) |  | 185 | 149 | 80.5  (74.1–86.0) | Reference  0.86 (0.38–1.96) |
|  | Rural | 55 | 28 | 50.9  (37.1–64.7) |  |  | 41 | 32 | 78.1  (62.4–89.4) |  |
| **Total** | | **184** | **111** | **60.3**  **(52.9–67.5)** | **Reference*** |  | **226** | **181** | **80.1**  **(74.3–85.1)** | **2.65 (1.70–4.11)** |

* Odds ratios and its 95% confidence intervals (CI) comparing nares (reference) to skin samples.
