# Supplementary material for: Prevalence, species identification, and antibiotic resistance of Staphylococci in dogs visiting veterinary clinics in Vietnam
Source: PLoS One. 2025 Jul 24;20(7):e0328472. doi: 10.1371/journal.pone.0328472 (PMC12289047; doi:10.1371/journal.pone.0328472)
Supplement: S5 Table — (DOCX) [file pone.0328472.s007.docx]

# S5 Table.

# Number and percentage of *Staphylococcus* isolates showing antibiotic resistance by health status and anatomical locations.

| **Antibiotics** | | **Diseased (n=227)** | **Healthy (n=82)** | ***p-*value** | **Nares (N=117)** | **Skin (N=192)** | ***p-*value** | **Total (N=309)** |
| --- | --- | --- | --- | --- | --- | --- | --- | --- |
| **Class** | **Agent** | **n (%)** | **n (%)** |  | **n (%)** | **n (%)** |  | **n (%)** |
| Beta-lactams | Ax | 192 (84.6) | 56 (68.3) | 0.001* | 100 (85.5) | 148 (77.1) | 0.072 | 248 (80.3) |
|  | Pn | 195 (85.9) | 49 (59.8) | 0.000* | 97 (82.9) | 147 (76.6) | 0.184 | 244 (79.0) |
|  | Am | 188 (82.8) | 54 (65.9) | 0.001* | 98 (83.8) | 144 (75.0) | 0.070 | 242 (78.3) |
|  | Ox | 103 (45.4) | 28 (34.2) | 0.078 | 47 (40.2) | 84 (43.8) | 0.537 | 131 (42.4) |
|  | Cn | 50 (22.0) | 12 (14.6) | 0.152 | 21 (18.0) | 41 (21.4) | 0.468 | 62 (20.1) |
|  | Ac | 29 (12.8) | 7 (8.5) | 0.305 | 20 (17.1) | 16 (8.3) | 0.020* | 36 (11.7) |
|  | Cp | 27 (11.9) | 6 (7.3) | 0.250 | 10 (8.6) | 23 (12.0) | 0.343 | 33 (10.7) |
| Phenicols | Cl | 79 (34.8) | 24 (29.3) | 0.362 | 46 (39.3) | 57 (29.7) | 0.082 | 103 (33.3) |
| Quinolones | Ci | 79 (34.8) | 18 (22.0) | 0.032* | 41 (35.0) | 56 (29.2) | 0.280 | 97 (31.4) |
|  | Lv | 61 (26.9) | 16 (19.5) | 0.187 | 33 (28.2) | 44 (22.9) | 0.297 | 77 (24.9) |
| Lincosamides | cL | 107 (47.1) | 29 (35.4) | 0.066 | 52 (44.4) | 84 (43.8) | 0.905 | 136 (44.0) |
| Tetracycline | Te | 149 (65.6) | 43 (52.4) | 0.035* | 73 (62.4) | 119 (62.0) | 0.942 | 192 (62.1) |
|  | Dx | 68 (30.0) | 17 (20.7) | 0.109 | 28 (23.9) | 57 (29.7) | 0.272 | 85 (27.5) |
| Macrolide | Er | 122 (53.7) | 42 (51.2) | 0.695 | 66 (56.4) | 98 (51.0) | 0.359 | 164 (53.1) |
|  | Az | 118 (52.0) | 39 (47.6) | 0.492 | 65 (55.6) | 92 (47.9) | 0.193 | 157 (50.8) |
| Aminoglycoside | Ge | 76 (33.5) | 16 (19.5) | 0.018* | 41 (35.0) | 51 (26.6) | 0.114 | 92 (29.8) |
|  | Ak | 17 (7.5) | 3 (3.7) | 0.227 | 7 (6.0) | 13 (6.8) | 0.785 | 20 (6.5) |
| Sulfonamide – trimethoprim | Bt | 82 (36.1) | 17 (20.7) | 0.010* | 44 (37.6) | 55 (28.7) | 0.102 | 99 (32.0) |
| Glycopeptid | Va | 0 (0.0) | 0 (0.0) | – | 0 (0.0) | 0 (0.0) | – | 0 (0.0) |
| Oxazolidinone | Li | 0 (0.0) | 0 (0.0) | – | 0 (0.0) | 0 (0.0) | – | 0 (0.0) |

n: Number of antibiotic-resistant strains;

* Statistically significant with *p*-value ≤ 0.05.
