# Supplementary material for: Prevalence, species identification, and antibiotic resistance of Staphylococci in dogs visiting veterinary clinics in Vietnam
Source: PLoS One. 2025 Jul 24;20(7):e0328472. doi: 10.1371/journal.pone.0328472 (PMC12289047; doi:10.1371/journal.pone.0328472)
Supplement: S6 Table — (DOCX) [file pone.0328472.s008.docx]

# S6 Table.

# Number and percentage of *Staphylococcus* isolates showing antibiotic resistance.

| **Antibiotics** | | **Nares (N=117)** | | ***p-*value** | **Skin (N=192)** | | ***p*-value** |
| --- | --- | --- | --- | --- | --- | --- | --- |
|  |  | **Diseased (n=81)** | **Healthy (n=36)** |  | **Diseased (n=146)** | **Healthy (n=46)** |  |
| **Class** | **Agent** | **n (%)** | **n (%)** |  | **n (%)** | **n (%)** |  |
| Beta-lactams | Ax | 75 (92.5) | 25 (69.4) | 0.001* | 117 (80.1) | 31 (67.4) | 0.073 |
|  | Pn | 76 (93.8) | 21 (58.3) | 0.000* | 119 (81.5) | 28 (60.9) | 0.004* |
|  | Am | 74 (91.3) | 24 (66.7) | 0.001* | 114 (78.1) | 30 (65.2) | 0.079 |
|  | Ox | 37 (45.6) | 10 (27.8) | 0.137 | 66 (45.2) | 18 (39.1) | 0.469 |
|  | Cn | 17 (20.9) | 4 (11.1) | 0.198 | 33 (22.6) | 8 (17.4) | 0.452 |
|  | Ac | 17 (20.9) | 3 (8.3) | 0.079 | 12 (8.2) | 4 (8.7) | 1.000 |
|  | Cp | 8 (9.9) | 2 (5.6) | 0.715 | 19 (13.0) | 4 (8.7) | 0.432 |
| Phenicols | Cl | 36 (44.4) | 10 (27.8) | 0.105 | 43 (29.5) | 14 (30.4) | 0.899 |
| Quinolones | Ci | 35 (43.2) | 6 (16.7) | 0.005* | 44 (30.1) | 12 (26.1) | 0.598 |
|  | Lv | 27 (33.3) | 6 (16.7) | 0.041* | 34 (23.3) | 10 (21.7) | 0.828 |
| Lincosamides | cL | 40 (49.3) | 12 (33.3) | 0.114 | 67 (45.9) | 17 (37.0) | 0.287 |
| Tetracycline | Te | 53 (65.4) | 20 (55.6) | 0.301 | 96 (65.8) | 23 (50.0) | 0.055 |
|  | Dx | 20 (24.6) | 8 (22.2) | 0.971 | 48 (32.9) | 9 (19.6) | 0.085 |
| Macrolide | Er | 52 (64.2) | 14 (38.9) | 0.017* | 70 (47.9) | 28 (60.9) | 0.126 |
|  | Az | 50 (61.7) | 15 (41.7) | 0.067 | 68 (46.6) | 24 (52.2) | 0.507 |
| Aminoglycoside | Ge | 34 (41.9) | 7 (19.4) | 0.034* | 42 (28.8) | 9 (19.6) | 0.218 |
|  | Ak | 6 (7.41) | 1 (2.8) | 0.661 | 11 (7.5) | 2 (4.3) | 0.737 |
| Sulfonamide – trimethoprim | Bt | 39 (48.1) | 5 (13.9) | 0.000* | 43 (29.5) | 12 (26.1) | 0.660 |
| Glycopeptid | Va | 0 (0.00) | 0 (0.00) | - | 0 (0.0) | 0 (0.0) | - |
| Oxazolidinone | Li | 0 (0.00) | 0 (0.00) | - | 0 (0.0) | 0 (0.0) | - |

* Statistically significant with *p*-value ≤ 0.05.
