# Supplementary material for: Prevalence, species identification, and antibiotic resistance of Staphylococci in dogs visiting veterinary clinics in Vietnam
Source: PLoS One. 2025 Jul 24;20(7):e0328472. doi: 10.1371/journal.pone.0328472 (PMC12289047; doi:10.1371/journal.pone.0328472)
Supplement: S7 Table — (DOCX) [file pone.0328472.s009.docx]

# S7 Table.

# Number of multidrug resistant *Staphylococcus* isolates per species.

| **Species** | **n** | **No. of multidrug resistance** | | **No. of MDR isolates**  **% (95% CI)** |
| --- | --- | --- | --- | --- |
|  |  | **Diseased**  **% (95% CI)** | **Healthy**  **% (95% CI)** |  |
| CoPS | 249 | 125/186  67.2 (59.9–73.9) | 40/63  63.5 (50.5–75.1) | 165  66.3 (60.0–72.1) |
| *S. aureus* | 25 | 15/22  68.2 (45.1–86.1) | 2/3  66.7 (9.4–99.1) | 17  68.0 (46.5–85.0) |
| *S. pseudintermedius* | 154 | 87/120  72.5 (63.6–80.2) | 21/34  61.8 (43.5–77.8) | 108  70.1 (62.2–77.2) |
| Other *Staphylococcus* | 70 | 23/44  52.3 (36.6–67.5) | 17/26  65.4 (44.3–82.7) | 40  57.1 (44.7–68.9) |
| CoNS | 60 | 15/41  36.6 (22.1–53.0) | 7/19  36.8 (16.2–61.6) | 22  36.7 (24.5–50.1) |
| *S. epidermidis* | 3 | 2/3  66.7 (9.4–99.1) | 0 | 2  66.7 (9.4–99.1) |
| *S. schleiferi* | 9 | 2/9  22.2 (2.8–60.0) | 0 | 2  22.2 (2.8–60.0) |
| Other *Staphylococcus* | 48 | 11/29  37.9 (20.7–57.7) | 7/19  36.8 (16.2–61.6) | 18  37.5 (23.9–52.6) |
| Total | 309 | 140/227  61.7 (55.0–68.0) | 47/82  57.3 (45.9–68.2) | 187  60.5 (54.8–66.0) |

CoPS: Coagulase–positive *Staphylococcus*; CoNS: Coagulase–negative *Staphylococcus*.
