# Supplementary material for: Prevalence, species identification, and antibiotic resistance of Staphylococci in dogs visiting veterinary clinics in Vietnam
Source: PLoS One. 2025 Jul 24;20(7):e0328472. doi: 10.1371/journal.pone.0328472 (PMC12289047; doi:10.1371/journal.pone.0328472)
Supplement: S8 Table — (DOCX) [file pone.0328472.s010.docx]

# S8 Table.

# Risk factors associated with MDR *Staphylococcus* spp.

| **Risk factors** | | **No. of isolate** | **No. of**  **MDR isolates** | **Percentage (%)**  **(95% CI)** | **OR**  **(95% CI)** |
| --- | --- | --- | --- | --- | --- |
| Health status | Diseased | 227 | 140 | 61.7 (55.0–68.0) | Reference |
|  | Healthy | 82 | 47 | 57.3 (45.9–68.1) | 0.83 (0.5–1.39) |
| Isolation site | Nares | 117 | 73 | 62.4 (52.9–71.1) | Reference |
|  | Skin | 192 | 114 | 59.4 (52.0–66.3) | 0.88 (0.55–1.41) |
| Species | *S. aureus* | 25 | 17 | 68.0 (46.5–85.0) | Reference |
|  | *S. pseudintermedius* | 154 | 108 | 70.1 (62.2–77.2) | 1.1 (0.45–2.74) |
|  | *S. epidermidis* | 3 | 2 | 66.7 (9.4–99.1) | 0.94 (0.07–11.97) |
|  | *S. schleiferi* | 9 | 2 | 22.2 (2.8–60.0) | 0.13 (0.02–0.8) |
|  | Others *Staphylococcus* | 118 | 58 | 49.2 (39.8–58.5) | 0.45 (0.18–1.14) |
| Prior use of antibiotics within 3 months | Yes | 120 | 104 | 86.7 (79.2–92.1) | Reference |
|  | No | 189 | 83 | 43.9 (36.7–51.3) | 0.12 (0.07–0.22) |
| Breed | Domestic | 85 | 47 | 55.3 (44.4–66.0) | Reference |
|  | Foreign | 224 | 140 | 62.5 (55.8–68.8) | 1.35 (0.81–2.23) |
| Gender | Male | 159 | 96 | 60.4 (52.3–68.0) | Reference |
|  | Female | 150 | 91 | 60.7 (52.3–68.5) | 1.01 (0.64–1.60) |
| Age (years) | <1 | 49 | 34 | 69.4 (54.5–81.7) | Reference |
|  | 1–5 | 144 | 79 | 54.9 (46.3–63.1) | 0.54 (0.27–1.07) |
|  | >5 | 116 | 74 | 63.8 (54.3–72.5) | 0.78 (0.38–1.59) |
| Management practices | Free–ranging | 74 | 46 | 62.2 (50.1–73.1) | Reference |
|  | Semi–confined | 137 | 88 | 64.2 (55.6–72.2) | 1.09 (0.61–1.96) |
|  | Confined | 98 | 53 | 54.1 (43.7–64.2) | 0.72 (0.39–1.33) |
| Location | Urban | 246 | 148 | 60.2 (53.7–66.3) | Reference |
|  | Rural | 63 | 39 | 61.9 (48.8–73.8) | 1.08 (0.61–1.9) |
| **Total** | | **309** | **187** | **60.5 (54.8–66.0)** |  |
