# Supplementary material for: Prevalence, species identification, and antibiotic resistance of Staphylococci in dogs visiting veterinary clinics in Vietnam
Source: PLoS One. 2025 Jul 24;20(7):e0328472. doi: 10.1371/journal.pone.0328472 (PMC12289047; doi:10.1371/journal.pone.0328472)
Supplement: S9 Table — (DOCX) [file pone.0328472.s011.docx]

# S9 Table.

# Number and percentage of *Staphylococcus* isolates showing antibiotic-resistance genes by health status and anatomical locations.

| **Class** | **Gene** | **Diseased (n=227)** | **Healthy (n=82)** | ***p-*value** | **Nares (N=117)** | **Skin (N=192)** | ***p-*value** | **Total**  **(N=309)** |
| --- | --- | --- | --- | --- | --- | --- | --- | --- |
|  |  | **n (%)** | **n (%)** |  | **n (%)** | **n (%)** |  | **n (%)** |
| Aminoglycoside | *aacA–aphD* | 159 (70.0) | 41 (50.0) | 0.001^*^ | 65 (55.6) | 135 (70.3) | 0.008^*^ | 200 (64.7) |
| Tetracycline | *tetK* | 121 (53.3) | 39 (47.6) | 0.372 | 63 (53.8) | 97 (50.5) | 0.570 | 160 (51.8) |
| Fluroquinolone | *gyrA* | 54 (23.8) | 33 (40.2) | 0.005^*^ | 32 (27.4) | 55 (28.6) | 0.806 | 87 (28.2) |
| Beta–lactam | *mecA* | 65 (28.6) | 17 (20.7) | 0.165 | 37 (31.6) | 45 (23.4) | 0.114 | 82 (26.5) |
| Macrolide | *mrsA* | 45 (19.8) | 35 (42.7) | 0.000^*^ | 37 (31.6) | 43 (22.4) | 0.072 | 80 (25.9) |
| Trimethoprim | *dfrA* | 14 (6.2) | 11 (13.4) | 0.039^*^ | 17 (14.5) | 8 (4.2) | 0.001^*^ | 25 (8.1) |
| Erythromycin | *ermA* | 3 (1.3) | 3 (3.7) | 0.192 | 3 (2.6) | 3 (1.6) | 0.536 | 6 (1.9) |
| Not detected^†^ | | 24 (10.6) | 14 (17.1) | 0.124 | 14 (12.0) | 24 (12.5) | 0.890 | 38 (12.3) |

n: Number of *Staphylococcus* spp. strains detected antibiotic-resistance genes;

^†^Not detect all 7 genes *aacA–aphD, tetK, gyrA, mecA, msrA, dfrA,* and *ermA*;

* Statistically significant with *p*-value ≤ 0.05.
